# Overexpression of RUNX2 promotes breast cancer multi-organ metastasis through stabilizing c-Myc

**DOI:** 10.1038/s41419-025-08018-9

**Published:** 2025-10-06

**Authors:** Tian-Hao Zhou, Hao Fu, Shuai Zhao, Wen-Jing Jiang, Sen Miao, Hao Tan, Rui Zhang, Qing-Shan Wang, Yu-Mei Feng

**Affiliations:** 1 Department of Biochemistry and Molecular Biology, Tianjin Medical University Cancer Institute and Hospital, National Clinical Research Center of Cancer, Tianjin, China; 2 Department of Pathology, Affiliated Hospital of Jining Medical University, Jining, China; 3 Key Laboratory of Breast Cancer Prevention and Therapy of the Ministry of Education, Tianjin, China

**Keywords:** Breast cancer, Breast cancer

## Abstract

Distant metastasis is the leading cause of mortality in breast cancer patients and remains a significant challenge in clinical practice. Although breast cancer metastasis exhibits organotropism, widespread dissemination and synchronous multi-organ metastasis frequently occur in advanced stages, or the early stages of patients suffering from aggressive tumors, even in patients with an undetectable primary tumor. However, the underlying mechanism is still far from being fully understood. Runt-related transcription factor 2 (RUNX2), a master osteogenic transcription factor, is commonly considered a driver of bone-specific metastasis in breast cancer. Surprisingly, we found here that overexpression of RUNX2 drives synchronous multi-organ metastases rather than bone-preferred metastasis in multiple mouse models of breast cancer, regardless of subtype. Mechanistically, RUNX2 physically interacts with c-Myc oncoprotein to prevent FBXW7-mediated ubiquitination and degradation of c-Myc and coordinately activates the transcription and expression of c-Myc target genes, which elicit early progression and spontaneous dissemination from primary tumor mass, rapid engraftment, and unrestrained outgrowth of cancer cells in distant organs. Thus, our findings uncover a novel mechanism of multi-organ metastasis and highlight RUNX2**‒**c-Myc regulatory axis as a prognostic indicator and a therapeutic target for predicting and managing multi-organ metastatic breast cancer.

## Introduction

Distant metastasis is the leading cause of mortality in breast cancer patients and poses a persistent therapeutic challenge. Although breast cancer metastasis exhibits subtype-specific organotropism [1], with distinct preferences for certain organs such as bone [2], lung [3], liver [4], and brain [5], synchronous multi-organ metastasis frequently occurs in advanced stages, or in the early-stage of patients suffering from aggressive tumors, even in patients with undetectable primary tumor [6]. Patients with multi-organ metastasis have a shorter median survival than those with single-site metastasis [7, 8]. As the terminal stage of the disease, multi-organ metastasis accounts for the vast majority of breast cancer-related deaths [9]. Multi-organ metastatic cancers consist of cell populations in a state of arrested differentiation, posing the capacities for autonomous proliferation and self-renewal, which elicit early progression and spontaneous dissemination from the primary tumor mass, rapid engraftment, and unrestrained growth in distinct organs [6]. Unfortunately, the knowledge of multi-organ metastasis remains limited, and effective therapeutic strategies are still lacking. Uncovering the crucial mechanisms of synchronous multi-organ metastasis will facilitate the identification of therapeutic targets and the development of effective intervention strategies.

c-Myc oncoprotein is a nuclear phosphoprotein belonging to the basic helix-loop-helix (bHLH) transcription factor superfamily. c-Myc acts as a heterodimerization partner of Myc-associated protein X (MAX) to synergistically activate the transcription of genes involved in a wide range of cell processes. Nearly all known mitogenic pathways ultimately stimulate *MYC* gene expression [10]. The principal function of c-Myc appears to drive quiescent cells into the cell cycle, prevent exit from the cell cycle, block terminal differentiation, and enhance metabolism of multiple cell types [10]. Therefore, elevated c-Myc expression drives oncogenic transformation of normal cells and plays a causal role in tumorigenesis. Additionally, c-Myc functions as an angiogenic switch that is essential for the progression and metastasis of tumors [11]. Overall, c-Myc is sufficient to function as a dominator for governing synchronous multi-organ metastasis in various types of cancers. However, how c-Myc is hijacked by upstream modulators and whether such an aberrant regulatory axis drives multiple-organ metastasis in different breast cancer subtypes remains to be investigated.

Runt-related transcription factor 2 (RUNX2) belongs to the Runx transcription factor family and is indispensable for skeletal development and remodeling through orchestrating a wide array of bone-related gene expression [12]. RUNX2 is also expressed in mammary epithelial cells [13] and is an essential regulator of epithelial cell fate in mammary gland development and breast cancer progression [14, 15]. It is known that RUNX2 protein is highly expressed in triple-negative breast cancers (TNBC) than in estrogen receptor-positive (ER+) breast cancers, and patients with high RUNX2 expression levels have poorer prognoses than those with low or undetectable expression levels. Elevated RUNX2 expression perturbs differentiation in the mouse mammary gland and renders the mammary epithelium more susceptible to oncogenic transformation in aged *Runx2* transgenic animals [16]. In addition, overexpression of *Runx2* drives epithelial-to-mesenchymal transition (EMT)-like changes in normal mammary epithelial cells, while *Runx2* deletion significantly increases tumor latency, reduces proliferation, and extends overall survival in a mouse model of luminal breast cancer (LumBC) [14]. These in vivo findings imply that overexpression of RUNX2 elicits a wide spectrum of malignant cellular behaviors in differentiation perturbation, tumorigenesis, cell proliferation, and EMT. Because RUNX2 regulates an array of gene expression involved in bone remodeling and cancer metastasis, it is constantly regarded as a driver of bone-specific metastasis in breast cancer [17]. However, conclusive clinical and experimental evidence supporting such a role of RUNX2 in breast cancer is still lacking.

In this study, we clarified the role of RUNX2 in regulating breast cancer metastasis via a series of animal experiments and clinical analyses. We surprisingly found that overexpression of RUNX2 promotes synchronous multi-organ metastasis in both ER+/LumBC and TNBC/basal-like breast cancer (BLBC). A mechanistic study on RUNX2-driven multi-organ metastasis uncovered that RUNX2 directly interacts with c-Myc to block its ubiquitination and degradation by hindering the binding of E3 ubiquitin ligase F-box/WD repeat-containing protein 7 (FBXW7), thereby stabilizing and elevating c-Myc oncoprotein.

## Methods

### Cell culture

The human breast cancer cell lines MCF-7 (RRID: CVCL_0031), T-47D (RRID: CVCL_0553), MDA-MB-231 (RRID: CVCL_0062) and BT-549 (RRID: CVCL_1092), the mouse breast cancer 4T1 cell lines (RRID: CVCL_0125) and EMT6 (RRID: CVCL_1923), and the human embryonic kidney 293T cell line (RRID: CVCL_0063) were obtained from American Type Culture Collection (Manassas, VA, USA) and cultured in Dulbecco’s Modified Eagle’s Medium (DMEM; MCF-7, MDA-MB-231, BT-549 and 293T) or RPMI 1640 (T-47D, 4T1 and EMT6), supplemented with 10% fetal bovine serum (FBS; Thermo Fisher Scientific/Gibco, Gaithersburg, MD, USA), 100 U/mL penicillin, and 100 µg/mL streptomycin (Invitrogen, Gaithersburg, MD, USA). All the cell lines were authenticated by short tandem repeat profiling and routinely tested for mycoplasma contamination.

### Generation of gene-overexpressing and knockdown cells

For gene overexpression, full-length cDNAs encoding human (CCDS43467.2) and mouse (CCDS37624.2) RUNX2, as well as the constructs encoding truncations of human RUNX2 C-terminal (ΔC-terminal), Runt domain (ΔRunt) domain, and N-terminal (ΔN-terminal) were cloned into the pLenti-3×Flag-Puro vector (Addgene, Massachusetts, USA). Full-length cDNAs encoding human (CCDS6359.2) and mouse (CCDS27504.1) c-Myc, along with constructs encoding truncations of Myc homology box (MB) 1 (ΔMB1), MB2 (ΔMB2), and MB3 (ΔMB3), were cloned into the PCDH-3×HA-Puro vector (Addgene). For gene knockdown, small hairpin RNAs (shRNAs) targeting RUNX2 or FBXW7 and a negative control shRNA were cloned into pLKO.1-EGFP-puro vector (Tsingke Co., Ltd, Beijing, China). To establish cells with stable gene overexpression or knockdown, lentiviral particles were generated in 293T cells by co-transfection of gene overexpression or knockdown plasmids with psPAX2 (RRID: Addgene_12260) and pMD2.0G (RRID: Addgene_12259) viral packaging vectors using Lipofectamine 2000 (Thermo Fisher Scientific), then infected cancer cells. All the infected cells were selected using 2 µg/mL puromycin (MedChemExpress, MCE; Shanghai, China) for 2 weeks. Small interfering RNAs (siRNAs) targeting E3 ubiquitin ligases involved in c-Myc ubiquitination were transfected into the cells using Lipofectamine 2000 following the manufacturer’s instructions. The sequences of shRNAs and siRNAs are listed in Table S1.

### RT‒qPCR

Total RNA of cells was extracted using TRIzol reagent (Thermo Fisher Scientific), and cDNA was synthesized using SuperScript™ First-Strand Synthesis System (TaKaRa, Dalian, China) following the manufacturer’s instructions. Quantitative PCR (qPCR) was performed using SYBR Green Mix (Vazyme, Nanjing, China) with primers listed in Table S2. The expression level of genes was quantified by normalizing the cycle threshold (Ct) values of the target gene to the Ct values of *GAPDH* (2^−ΔCT^).

### Immunoblot

Cells were lysed using a denatured lysis buffer (50 mM Tris-HCl, pH 7.4, 0.5 mM EDTA, 1% SDS, 1% DTT). Protein concentration was determined via ultraviolet spectrophotometry. Total protein of 40 μg was loaded for each sample and separated by SDS-PAGE, followed by being transferred onto nitrocellulose membranes. After blocking with 5% skimmed milk in TBST (TBS with 0.05% Tween-20), membranes were incubated with primary antibodies in 2% BSA in TBST at 4 °C overnight. Following three washes with TBST, the membranes were incubated with HRP-conjugated secondary antibodies in 5% skimmed milk in TBST, and the signals were visualized using ECL substrate (Vazyme) using Tanon imaging system (Tanon, Beijing, China). The detailed information of antibodies is listed in Supplementary Table 3. Uncropped blot images are presented in the supplementary material.

### Cell proliferation assays

Cells were suspended in 100 μL of complete medium at an appropriate density and seeded into 96-well plates in triplicate per group. At day 0, 1, 2, 3, 4, and 5 post-seeding, 10 μL Cell Counting Kit-8 (MCE) reagent was added to each well for 2 h at 37 °C, and then absorbance was measured at 450 nm using a BioTek Synergy (Thermo Fisher Scientific). Cell growth curves were generated based on the OD values at each culture time point.

### Cell cycle analysis

Cells in the logarithmic growth phase were digested with trypsin and fixed with 70% ethanol at 4 °C overnight. After two washes with cold PBS, cells were resuspended in 50 μg/mL propidium iodide (PI) containing RNase (Sigma–Aldrich, Missouri, USA) and incubated for 30 min at room temperature. The distribution of cell cycle phases was detected and analyzed using a CytoFLEX flow cytometer (Beckman, California, USA).

### Cell migration and invasion assays

Cells were resuspended in serum-free medium and seeded into the upper chamber of Transwell inserts (8.0 μm pore size; Corning, New York, USA), either uncoated (for migration assays) or coated (for invasion assays) with Matrigel (Corning). Complete culture medium was added to the lower chambers. After an appropriate period, non-migrated or non-invaded cells on the upper side of the membrane were removed, and migrated or invaded cells were fixed with 4% paraformaldehyde (PFA), stained with 0.5% crystal violet, and counted under a microscope in at least three random fields for each chamber at 200× magnification.

### Cell colony formation assay

Cells were seeded into 6-well plates at a density of 300 cells per well and cultured for an appropriate period. After being fixed with 4% PFA, the cells were stained with 0.5% crystal violet, and colonies with more than 50 cells were counted.

### Animal experiments

All animal experiments were approved and performed according to the guidelines and regulations of Tianjin Medical University Animal Care and Use Committee (Approval Number: TMUaMEC2023019). For the orthotopic xenograft tumor models, 1 × 10^7^ T-47D-Luc-GFP, 1 × 10^6^ MDA-MB-231-Luc-GFP, or 1 × 10^5^ 4T1-Luc-GFP cells with overexpression or knockdown of the indicated genes were suspended in PBS and injected into the fourth mammary fat pad of 4- to 6-week-old female NOD-SCID (T-47D and MDA-MB-231) or BALB/c (4T1) mice. The length (L) and width (W) of the primary tumors were measured using calipers, and tumor volume was calculated according to the formula: L × W^2^/2. For the hematogenous metastasis models, 1 × 10^6^ T-47D-Luc-GFP or 1 × 10^5^ MDA-MB-231-Luc-GFP cells with RUNX2 overexpression or corresponding empty vector were injected into the tail vein or left ventricle of 4–6-week-old female NOD-SCID mice. Animals of similar age and weight were randomly selected and grouped. The experimental period relies on the tumor burden and the physiological state of mice. At the endpoint of each experiment, 100 μL of luciferin (Promega, Wisconsin, USA) was injected retro-orbitally, and in vivo bioluminescence imaging (BLI) of anesthetized mice, as well as in vitro imaging of the bone, lung, liver, diaphragm, spleen, kidney, and brain, was performed using an IVIS Spectrum device (PerkinElmer, Massachusetts, USA; for BLI) or a Tanon system (for in vitro organ imaging). Visceral organs and hind limb bones were excised from euthanized mice and fixed in 4% PFA overnight. Bone tissues were decalcified in 10% EDTA for 2 weeks. Tissue embedding, slide sectioning, and H&E staining were performed following standard operating procedures.

### Flow cytometric analysis

Single cells were isolated from peripheral blood, bone marrow, and lung tissues of mice and resuspended in PBS. The number of GFP-labeled cancer cells was detected using a CytoFLEX flow cytometer. The GFP-labeled cancer cell counts per million peripheral blood, BM, or lung tissue cells were analyzed and presented using FlowJo V10 software (BD, New Jersey, USA).

### RNA sequencing

Total RNA from three parallel samples of MDA-MB-231 cells with stable knockdown of RUNX2 and the control was extracted using TRIzol reagent according to the manufacturer’s instructions. Non-strand-specific paired-end sequencing libraries were generated with TruSeq Stranded mRNA and sequenced on the Illumina NovaSeq platform. Reads were mapped to the human reference genome GRCh38 with the Broad Picard Pipeline. Gene expression levels were estimated with GenomicAlignments (v.1.18.1), and differential analysis was performed using DESeq2 (v.1.24.0). Gene set enrichment analysis (GSEA) was performed to calculate enrichment scores for hallmark gene sets based on the normalized read counts using GSEA4.3.2 software (http://www.broadinstitute.org/gsea/index.jsp). Differentially expressed genes were filtered using an adjusted *P* < 0.05 and a fold change of less than 0.66 or greater than 1.5. Kyoto Encyclopedia of Genes and Genomes (KEGG) pathway enrichment was analyzed based on the downregulated genes (DRGs) using the Database for Annotation, Visualization, and Integrated Discovery system. For GSEA of the MYC target genes and related signaling pathways, the HALLMARK_MYC_TARGETS_V1, HALLMARK_MYC_TARGETS_V2, KEGG_CELL_CYCLE, and KEGG_ALANINE, ASPARTATE_AND_GLUTMATE_METABOLISM gene sets were analyzed using GSEA4.3.2 software. Gene sets were obtained from the Molecular Signatures Database (https://www.gsea-msigdb.org/gsea/msigdb/).

### Gene expression profiling and single-cell transcriptome datasets

The gene expression profiling dataset from The Cancer Genome Atlas Breast Cancer (TCGA-BRCA) project was used to validate the correlation between genes in primary breast cancer tissues (*n* = 1089). Smartseq2 single-cell RNA-sequencing (scRNA-seq) data of TNBC breast samples (GSE123837_HCI010) were obtained from Gene Expression Omnibus (GEO; http://www.ncbi.nlm.nih.gov/geo/) database. For the scRNA-seq data analysis, breast cancer cells characterized by the CD298^+^/MHC-I^−^ phenotype were analyzed to dissect the underlying association between *RUNX2* and *MYC,* as well as c-Myc target genes *CDK4*, *PCNA,* and *SLC1A5* at single-cell resolution. The FPKM value matrices from the GSE123837_HCI010 cohort were analyzed using the “Seurat” package in R with default parameters. For quality control, cells with gene expression levels lower than 2500 and those with mitochondrial gene expression exceeding 50% were excluded. Additionally, genes expressed in fewer than 8 cells were also removed.

### In vivo ubiquitylation assays

For exogenous c-Myc ubiquitylation detection, His-tagged Ubiquitin, Flag-RUNX2, HA-c-Myc plasmids, and corresponding controls were co-transfected into 293T cells and treated with 10 μM MG-132 for 4 h. Cells were lysed with denatured buffer and boiled at 100 °C for 10 min. Samples were then diluted tenfold with wash buffer (50 mM Tris, 150 mM NaCl, 1 mM EDTA) and ubiquitinated proteins were purified using Ni-NTA beads. HA-tagged proteins were detected by immunoblot. For endogenous c-Myc ubiquitination detection, cells were lysed with denatured buffer and boiled at 100 °C for 10 min, and after treated with 10 μM MG-132 for 4 h. The samples were then diluted tenfold with immunoprecipitation (IP) lysis buffer (50 mM Tris-HCl, 150 mM NaCl, 0.05 mM EDTA, 1% NP40, and 10% glycerol) and incubated overnight with an anti-c-Myc antibody. Total ubiquitinated proteins were detected by immunoblot. The detailed information on antibodies is listed in Table S3.

### IP analysis

The cells transfected with the indicated vector were washed with ice-cold PBS and lysed in IP buffer. Cell lysates were clarified by centrifugation at 20,000 × *g* for 3 min at 4 °C. 5% of the total clarified lysate was stored as input, and the remaining samples were incubated with the corresponding antibodies overnight at 4 °C. Pre-washed protein A/G beads were then added into the IP samples and incubated for 1 h at 4 °C. The supernatant was discarded, and the beads were washed 5 times with IP buffer. The immune complexes were detected using an immunoblot. The detailed information of antibodies is listed in Supplementary Table 3.

### Protein purification and in vitro Co-IP

For acquiring recombinant Flag-fused RUNX2 protein, full-length human *RUNX2* cDNA was cloned into the PET28a-3×Flag-Kan vector (Addgene). The Flag-fused RUNX2 construct was transformed into E. coli BL21 cells, and the bacterial cells were lysed using ultrasound, and the fused protein was purified by Flag antibody-coupled magnetic beads. His-fused c-Myc protein was purchased from Cusabio Co., Ltd (Wuhan, China). For the His pull-down assay, 2 μg of the His-fused c-Myc, Flag-fused RUNX2, and Ni-NTA beads were mixed with 50 μL IP buffer containing protease inhibitor cocktail and rotated at 4 °C for 6 h. Beads were washed five times with IP buffer and then resuspended with 1× SDS-PAGE loading buffer. The fused proteins were detected by Coomassie staining and immunoblot.

### ChIP-sequencing (ChIP–seq) and CUT&RUN-sequencing (CUT&RUN–seq) data analyses

The promoter regions of c-Myc target genes enriched by ChIP–seq using an anti-c-Myc antibody (GSE95303) and CUT&RUN–seq using an anti-RUNX2 antibody (GSE190248) in MDA-MB-231 cells were obtained from the GEO database. The data were visualized using the Integrative Genomics Viewer (IGV) tool (https://igv.org/).

### Re-ChIP-qPCR

The *CDK4*, *PCNA,* and *SLC1A5* promoter regions containing c-Myc binding elements were enriched using an anti-c-Myc or anti-RUNX2 antibody in T-47D cells with RUNX2 overexpression and MDA-MB-231 cells. The anti-c-Myc or anti-RUNX2 antibody-enriched promoter regions of the target genes were used to perform Re-ChIP with an anti-RUNX2 or anti-c-Myc antibody, as previously described [18]. All antibodies and their dilutions used in ChIP assays are listed in Table S3. The enriched DNA fragments were detected by qPCR using the primers listed in Table S4. The quantity of ChIP-enriched DNA fragments was calculated as a percentage of input.

### Patients and tissue specimens

RUNX2 and c-Myc protein levels in breast cancer tissue specimens were analyzed by immunohistochemistry of formalin-fixed, paraffin-embedded (FFPE) tissues. The specimens included 132 primary, 8 bone metastatic, and 11 visceral-metastatic breast cancer tissues. All the samples and follow-up data acquisition procedures were under the approval, and all the methods were performed in accordance with the guidelines of the Human Ethics Committee of Jining Medical University Affiliated Hospital, Jining Medical University (Approval Number: 2024-04-C10). Written informed consent was obtained from all of the patients. The baseline clinicopathological characteristics of patients are listed in Table 1.

**Table 1:** Clinical and pathological characteristics of breast cancer patients.

Characteristic	Non-Met. (%)	Met. (%)	*P* (*χ*^2^)
**Age**			0.1059
≤50	46 (70.7)	19 (29.3)	
>50	38 (58.4)	29 (41.6)	
**ER status**			0.1482
Positive	53 (68.8)	24 (31.2)	
Negative	31 (56.4)	24 (43.6)	
**PR status**			0.0707
Positive	46 (71.9)	18 (28.1)	
Negative	38 (55.9)	30 (44.1)	
**HER2 status**			0.1809
Unamplified	53 (59.6)	36 (40.4)	
Amplified	31 (72.1)	12 (27.9)	
**Ki-67**			0.3857
Low	41 (63.1)	20 (33.9)	
High	37 (60.7)	25 (67.6)	
Missing	0 (0.0)	3 (100.0)	
**Molecular subtype**			0.3857
Luminal A	21 (80.8)	5 (19.2)	
Luminal B	29 (61.7)	18 (38.3)	
HER2 enriched	15 (88.2)	2 (11.8)	
TNBC/BLBC	19 (45.2)	23 (54.8)	
**Pathologic grade**			0.0004
1‒2	51 (78.5)	14 (21.5)	
3	32 (48.5)	34 (51.5)	
**Tumor stage**			0.0411
T1	14 (66.7)	7 (33.3)	
T2‒3	60 (60.6)	39 (39.1)	
T3	0 (0.0)	4 (100.0)	
**Node stage**			0.0727
N0	44 (73.3)	16 (26.7)	
N1	19 (61.3)	12 (38.7)	
N2‒3	21 (51.2)	20 (48.8)	

*Met* metastasis, *ER* estrogen receptor, *PR* progesterone receptor, *HER2* human epidermal growth factor receptor 2, *TNBC/BLBC* triple-negative breast cancer/basal-like breast cancer.

### IHC and mIHC

Immunohistochemistry (IHC) assays were performed as described previously [19]. The IHC score (values 0–12) was calculated by multiplying the staining intensity score by the frequency of positive staining cells score in tumor cells. Staining intensity was assessed manually using scores of 0 (negative), 1 (weak), 2 (moderate), and 3 (strong). The frequencies of positively stained cells were assessed manually using scores of 0 (<5%), 1 (5%–25%), 2 (26%–50%), 3 (51%–75%), 4 (>75%). For multiplex IHC (mIHC), FFPE tissue sections were performed using the Opal 6-Plex Manual Detection Kit (NEL861001KT, Akoya, Delaware, USA) according to the manufacturer’s instructions and as described previously [1]. Briefly, the sections were deparaffinized, rehydrated, subjected to antigen retrieval (high pressure for the first cycle and microwave for the following cycle), and blocked. Then, the sections were incubated with primary antibodies overnight at 4 °C. After 3 washes with TBST and incubation with an HRP-conjugated secondary antibody, the signals were visualized using Opal-labeled tyramide signal amplification. The sections were subjected to 3 sequential cycles of the above procedures. Nuclei were stained with DAPI. Whole-slide images were scanned using a Vectra Polaris scanner (Akoya), and the signals were analyzed using Phenochart software (Akoya). The detailed information of antibodies is listed in Table S3.

### Statistical analysis

All the in vitro experiments were performed at least two independent times in triplicate, and data are presented as the mean ± SD. Details of data presentation and statistical methods are described in the figure legends. Differences with *P* < 0.05 were considered statistically significant. All analyses were performed using GraphPad Prism 6.0 Software (GraphPad, La Jolla, CA, USA).

## Results

### RUNX2 promotes breast cancer cell proliferation and multi-organ metastasis

To investigate the role of RUNX2 in breast cancer proliferation and metastatic potential in vitro, we stably overexpressed RUNX2 in LumBC MCF-7 and T-47D cells (low RUNX2 expression) and knocked down RUNX2 in BLBC MDA-MB-231 and 4T1 cells (high RUNX2 expression) via lentiviral infection (Fig. S1A–E). CCK-8 and colony formation assays showed that RUNX2 overexpression promoted MCF-7 and T-47D cell proliferation, while silencing RUNX2 inhibited the proliferation of MDA-MB-231 and 4T1 cells (Fig. S2A, B). Furthermore, cell cycle assays revealed that RUNX2 overexpression promoted cell cycle progression into the S and G2/M phases, while RUNX2 knockdown led to an accumulation of cells in the G0/G1 phase and a reduction in the S phase and G2/M phase (Fig. S2C). Additionally, Transwell assays demonstrated that RUNX2 positively regulated the migration and invasion abilities of breast cancer cells (Fig. S2D). Collectively, these results suggest that RUNX2 promotes breast cancer cell proliferation and metastatic potential.

**Fig. 1:**
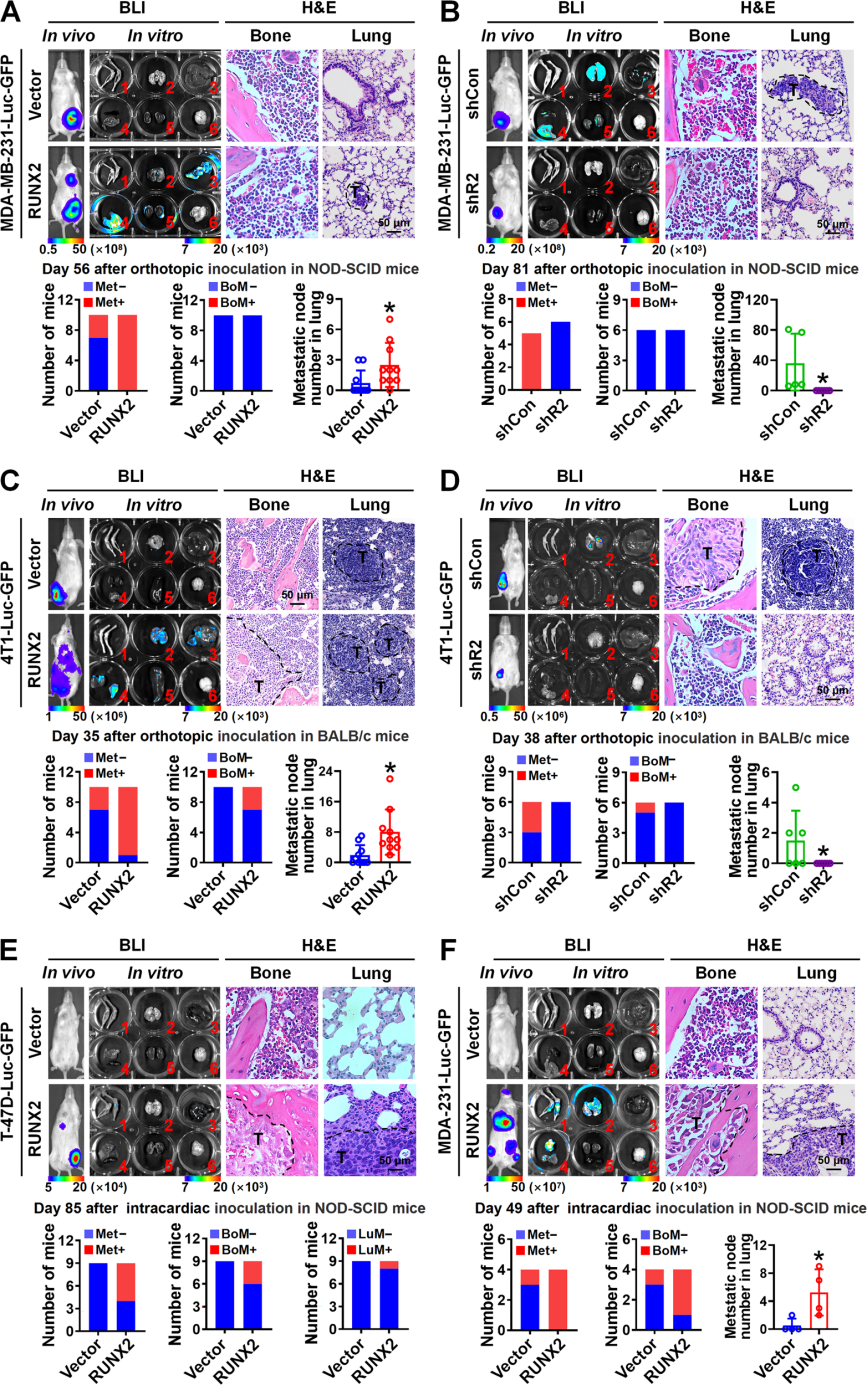
RUNX2 promotes multi-organ metastasis of breast cancer in vivo. **A**–**D** Breast cancer cells were orthotopically inoculated into mice, including MDA-MB-231-Luc-GFP cells (1 × 10^6^ cells per NOD-SCID mouse) with stable RUNX2 overexpression (*n* = 10 mice per group, sacrificed on day 56) or knockdown (*n* = 6 mice per group, sacrificed on day 81), and 4T1-Luc-GFP cells (1 × 10^5^ cells per BALB/c mouse) with stable RUNX2 overexpression (*n* = 10 mice per group, sacrificed on day 35) or knockdown (*n* = 6 mice per group, sacrificed on day 38) as well as corresponding controls. **E** and **F** Breast cancer cells were intracardially inoculated into the NOD-SCID mice, including T-47D-Luc-GFP cells (1 × 10⁶ cells per mouse, *n* = 9 per group, sacrificed on day 85) and MDA-MB-231-Luc-GFP cells (1 × 10^5^ cells per mouse, *n* = 4 per group, sacrificed on day 49) with RUNX2 overexpression or vector control. Representative images of in vivo and in vitro BLI (1 = bone, 2 = lung, 3 = liver, 4 and 5 = spleen, diaphragm, spleen and kidney, and 6 = brain) as well as H&E staining of the bones and lungs are shown. The number of mice with metastasis detected by BLI, bone metastasis, and lung-metastatic nodes verified by H&E were counted in each group. Data are presented as mean ± SD. Statistical analyses were performed with the unpaired Student’s *t*-test. *P < 0.05 compared with the control. BLI bioluminescence imaging, shControl shControl, shR2 shRUNX2. BoM bone metastasis, LuM lung metastasis.

To investigate the roles of RUNX2 in breast cancer proliferation and metastasis in vivo, LumBC T-47D-Luc-GFP cells (low tumor-initiating and lacking spontaneously metastatic potential) with RUNX2 overexpression, BLBC MDA-MB-231-Luc-GFP cells (high tumor-initiating and spontaneously metastatic potential) with RUNX2 overexpression or knockdown, as well as control cells, were orthotopically inoculated into immunodeficient NOD-SCID mice. T-47D cells overexpressing RUNX2 exhibited greater tumor-initiating capacity and formed larger tumor volumes (Fig. S3A). RUNX2 overexpression in MDA-MB-231 cells also promoted orthotopic tumor growth (Figs. 1A and S3B). Moreover, RUNX2 overexpression significantly promoted MDA-MB-231 cells to metastasize to multiple organs, including the lung, liver, diaphragm, and kidney, which were detected using BLI and verified by H&E staining (Figs. 1A and S3C). Conversely, RUNX2 knockdown in MDA-MB-231 cells suppressed tumor initiation and growth abilities (Fig. S3D), and diminished metastases to visceral organs (Fig. 1B). It should be explained that due to different end time points of experiments, the metastatic events in the two control groups of MDA-MB-231 cells with Vector and shControl exhibited significant variation. Additionally, similar to parental MDA-MB-231 cells, both the RUNX2 overexpression and knockdown failed to exhibit bone metastatic potential when orthotopically inoculated. (Fig. 1A, B). We further orthotopically inoculated mouse-derived BLBC 4T1-Luc-GFP cells with RUNX2 overexpression or knockdown and their corresponding control into immunocompetent BALB/c mice. Consistently, RUNX2 positively regulated tumor growth and multi-organ metastasis in the orthotopic mouse models (Figs. 1C, D and S3E–G).

To further validate the promoting role of RUNX2 in breast cancer multi-organ metastasis, T-47D-Luc-GFP and MDA-MB-231-Luc-GFP cells with RUNX2 overexpression or corresponding control were intracardially or intravenously injected into NOD-SCID mice. Intracardial inoculation demonstrated that RUNX2 overexpression promoted both T-47D and MDA-MB-231 cells to metastasize to bone, lung, liver, and brain (Fig. 1E, F). Intravenous inoculation also demonstrated that RUNX2 overexpression increased lung-metastatic burden in both T-47D and MDA-MB-231 cells (Fig. S3H, I). Taken together, these results demonstrate that RUNX2 promotes breast cancer cell growth and multi-organ metastasis rather than bone-preferred metastasis in both LumBC and BLBC.

### RUNX2 boosts c-Myc signaling in breast cancer through elevating c-Myc protein level

**Fig. 2:**
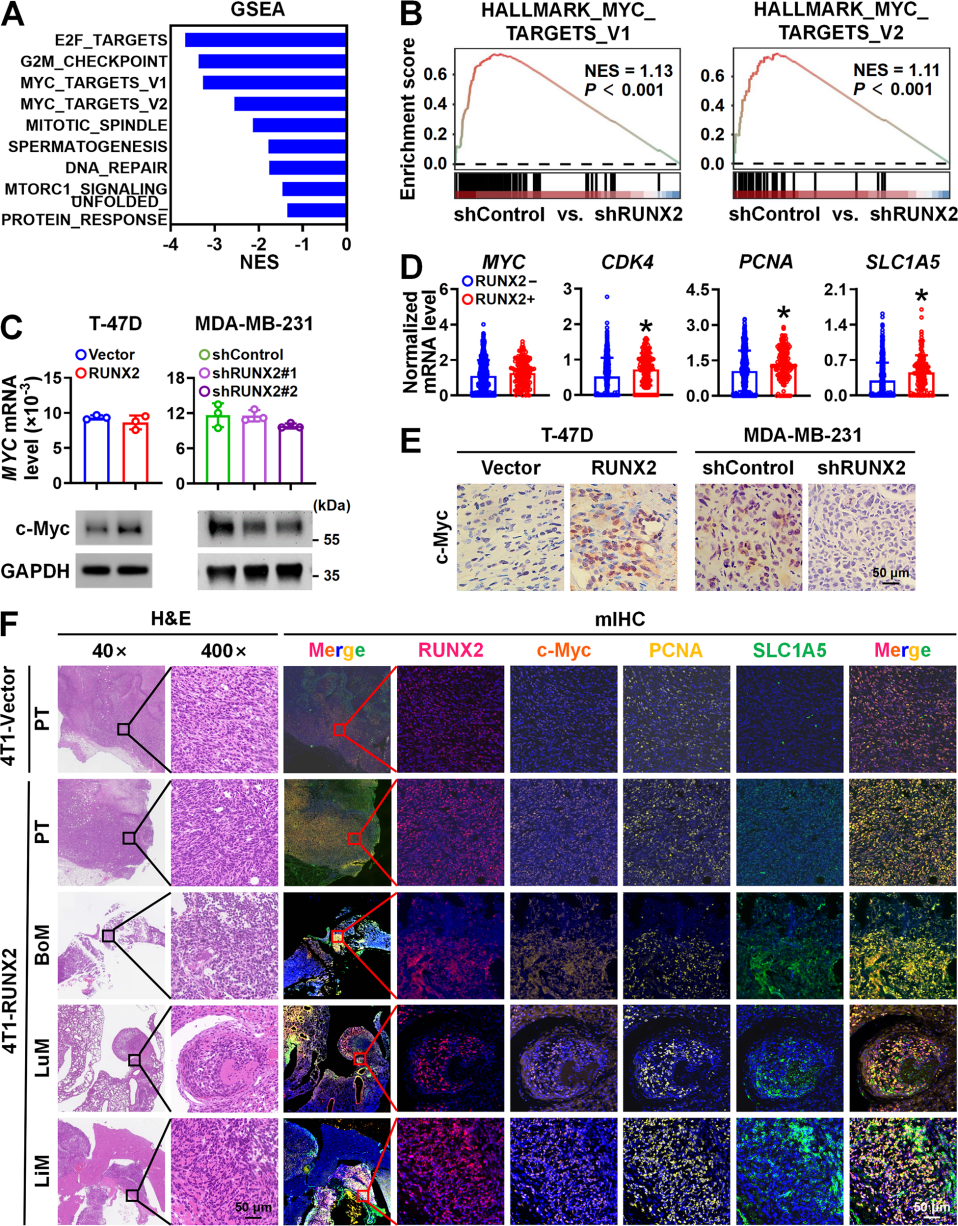
RUNX2 boosts c-Myc signaling in breast cancer cells through elevating c-Myc protein level. Transcriptomic analysis was conducted in MDA-MB-231 cells with RUNX2 knockdown and the control cells. Downregulated signal pathways by RUNX2 knockdown in MDA-MB-231 cells based on the GSEA of RNA-seq data (**A**). The gene sets of HALLMARK_MYC TARGET_GENE_V1 and V2 were significantly downregulated in RUNX2-knockdown MDA-MB-231 cells (**B**). **C** c-Myc mRNA and protein levels in the indicated cells were detected using RT–qPCR and immunoblot, respectively. **D** Comparison of the mRNA expression levels of the indicated genes in RUNX2-negative and RUNX2-positive breast cancer cells based on a breast cancer single-cell Smartseq2 dataset (GSE123837_HCI010). **E** c-Myc protein levels in the primary tumors formed by the indicated cells were detected by IHC. **F** RUNX2, c-Myc, PCNA, and SLC1A5 protein levels in the tissues of primary tumor (PT), bone metastasis (BoM), lung metastasis (LuM), and liver metastasis (LiM) formed by the indicated cells in orthotopic tumor models were detected by mIHC. Data are shown as the mean ± SD. Statistical analyses were performed with the unpaired Student’s *t*-test. *P < 0.05 compared with the control cells. mIHC multiplex immunohistochemistry.

To investigate the mechanism of RUNX2 in promoting multi-organ metastasis in breast cancer, we performed a transcriptomic analysis of MDA-MB-231 cells with RUNX2 knockdown and the control cells. The most prevalent downregulated gene sets upon RUNX2 silencing were E2F_TARGETS, G2/M_CHECKPOINT, MYC_TARGETS, DNA_REPAIR and MTORC1_SIGNALING, all of which are involved in cell cycle, proliferation, survival and metabolism (Fig. 2A). KEGG enrichment analysis of downregulated genes revealed that the most prevalent downregulated pathways were also involved in cell cycle, DNA replication, DNA repair, and various metabolism processes (Fig. S4A). c-Myc is a key regulator in promoting tumorigenesis and progression by enhancing cell cycle progression, facilitating DNA damage repair, and modulating metabolism [20], which highly coincides with the enriched pathways following RUNX2 knockdown. Therefore, we hypothesized that RUNX2 promotes breast cancer proliferation and metastasis by engaging the c-Myc signal pathway. Then, we analyzed the expression levels of c-Myc target genes and pathways in the two group cells. The results indicated that c-Myc target genes involved in cell cycle and amino acid metabolism processes were dramatically downregulated in RUNX2 knockdown cells (Figs. 2B and S4B). The heatmap of c-Myc target genes revealed that genes related to cell cycle, DNA damage repair, and metabolism were significantly downregulated in RUNX2 knockdown cells and were validated by RT–qPCR in the expression levels of *CDK4*, *PCNA*, and *SLC1A5* (Fig. S4C, D). Intriguingly, neither overexpression of RUNX2 in T-47D nor knockdown of RUNX2 in MDA-MB-231 affected *MYC* mRNA expression levels. In contrast, overexpression of RUNX2 in T-47D cells increased, while knockdown of RUNX2 in MDA-MB-231 reduced c-Myc protein levels (Fig. 2C). We next analyzed the correlation between *RUNX2* and c-Myc target genes, including *CDK4*, *PCNA,* and *SLC1A5*, in breast cancer tissues based on the TCGA-BRCA dataset. However, no significant correlations were observed (Fig. S4E). Given that RUNX2 is highly expressed in stromal cells, such as cancer-associated fibroblasts, endothelial cells, and immune cells, whereas *CDK4*, *PCNA,* and *SLC1A5* are predominantly expressed in epithelial cells (Fig. S4F), bulk RNA-seq of the entire tumor mass may not capture this potential relationship. To address this issue, we analyzed a breast cancer Smartseq2 scRNA-seq dataset (GSE123837_HCI010) and found that *RUNX2* expression was positively correlated with *CDK4*, *PCNA* and *SLC1A5* expression, but not with *MYC* in breast cancer cells (Fig. 2D). Consistently, c-Myc protein levels in the orthotopic xenograft tumor of T-47D and MDA-MB-231 cells were positively regulated by RUNX2 (Fig. 2E). Further, the protein levels of RUNX2, c-Myc, PCNA and SLC1A5 were evaluated using mIHC in primary tumor tissue formed by 4T1-vector and 4T1-RUNX2 cells, as well as in lung, liver, and bone metastasis tissues formed by 4T1-RUNX2 cells in orthotopic allograft tumor model. Elevated expression of c-Myc, PCNA, and SLC1A5 was observed in both primary and metastatic tumors overexpressing RUNX2 (Fig. 2F). Taken together, these results demonstrate that RUNX2 boosts c-Myc-mediated cell cycle progression and metabolism enhancement in breast cancer cells by elevating the protein level of c-Myc but not the mRNA level.

### RUNX2 directly interacts with c-Myc and stabilizes c-Myc

**Fig. 3:**
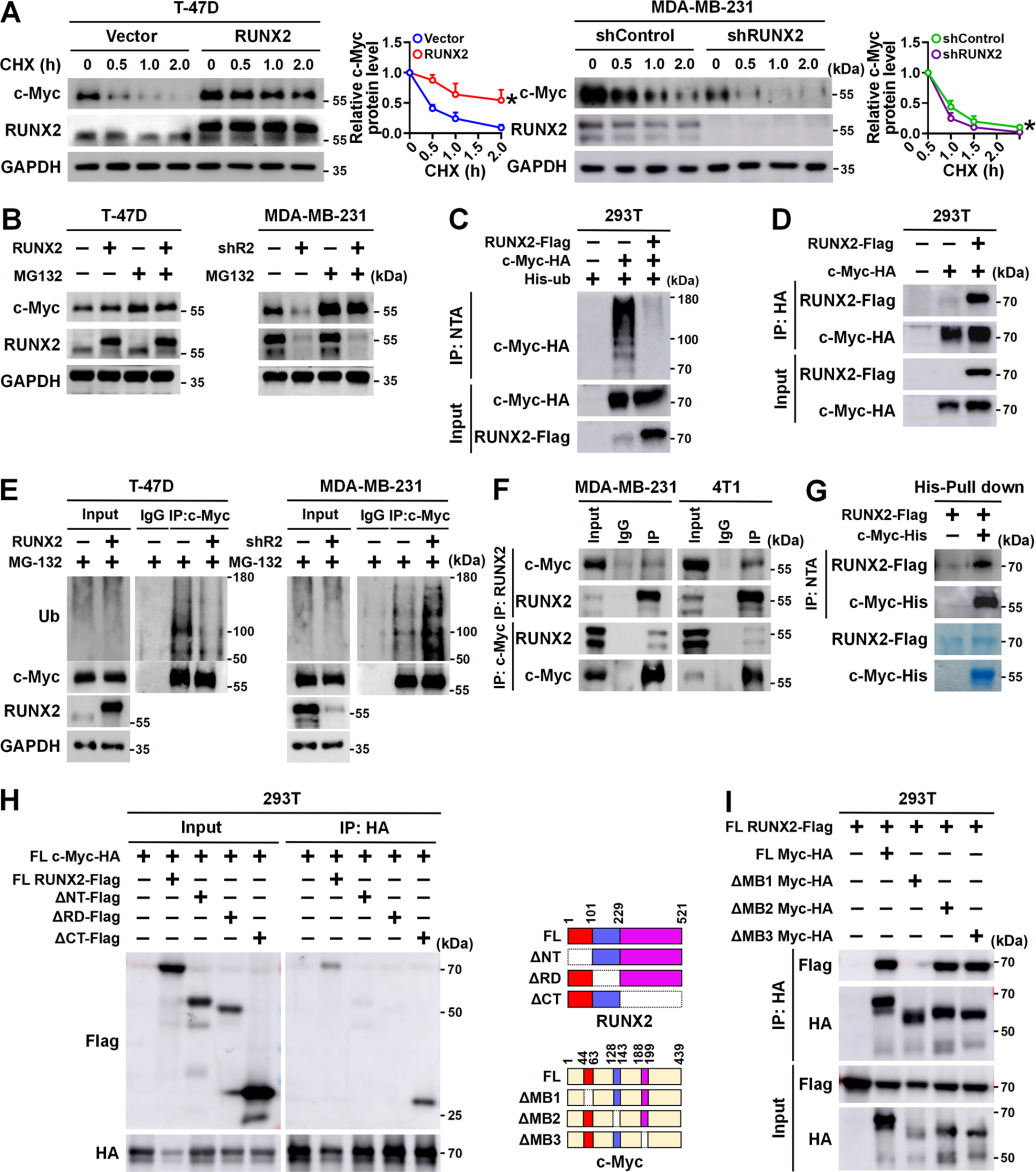
RUNX2 stabilizes c-Myc via direct protein interaction and forms a complex to cooperatively activate c-Myc target gene expression. **A** The impact of RUNX2 on the half-life of c-Myc in the indicated cells was evaluated by immunoblot. Relative c-Myc protein levels in the cells treated with CHX for different time points were quantified from three independent experiments. **B** T-47D cells with RUNX2 overexpression and MDA-MB-231 with RUNX2 knockdown were treated with 10 μM MG-132 for 4 h. c-Myc protein levels in the indicated cells were detected by immunoblot. **C** 293T cells were transiently co-transfected with c-Myc-HA and RUNX2-Flag or vector control for 48 h followed 4 h treatment with 10 μM MG-132. Ubiquitination levels of exogenous c-Myc in the indicated cells were detected using IP and immunoblot. **D** 293T cells were co-transfected with RUNX2-Flag and c-Myc-HA. Interaction between exogenous RUNX2 and c-Myc proteins was detected **E** T-47D cells with stable RUNX2 overexpression and MDA-MB-231 cells with stable RUNX2 knockdown, as well as the corresponding control cells were treated with 10 μM MG-132 for 4 h. Ubiquitination of endogenous c-Myc was analyzed using IP and immunoblot. **F** Interaction between endogenous RUNX2 and c-Myc proteins in MDA-MB-231 and 4T1 cells was detected using Co-IP and immunoblot. **G** His-fused c-Myc and Flag-fused RUNX2 proteins expressed in bacteria were purified and were used for the His pull-down assay. Protein interaction between the two proteins was detected using an immunoblot. Interaction of HA-tagged c-Myc with Flag-tagged full-length RUNX2 or its mutants lacking N-terminal (ΔNT), Runt-homology domain (ΔRD), or C-terminal (ΔCT) (**H**), as well as Flag-tagged RUNX2 with HA-tagged full-length c-Myc or its mutants lacking MB1 domain (ΔMB1), MB2 domain (ΔMB2), and MB3 domain (ΔMB3) (**I**) in 293T cells were detected using Co-IP and immunoblot. CHX cycloheximide, shR2 shRUNX2, IP immunoprecipitation, FL full length.

To gain insight into the mechanisms by which RUNX2 contributes to the elevation of c-Myc protein level, the stability of c-Myc was evaluated in T-47D and MDA-MB-231 cells following cycloheximide (CHX) treatment. The results showed that overexpression of RUNX2 in T-47D significantly prolonged, while knockdown of RUNX2 in MDA-MB-231 shortened the half-life of c-Myc protein (Fig. 3A). Since the ubiquitin–proteasome pathway is the most prominent mechanism for regulating c-Myc protein level [21], we investigated whether RUNX2 modulated c-Myc protein stability through the ubiquitin–proteasome pathway. We found that c-Myc protein levels robustly increased in breast cancer cells treated with MG-132, but were less affected by either overexpression or knockdown of RUNX2 in the presence of MG-132 (Fig. 3B). These results suggested that RUNX2 regulates c-Myc protein level depending on the ubiquitin–proteasome pathway. Furthermore, the ubiquitination of c-Myc was detected in the presence or absence of RUNX2 in 293T cells and the results confirmed that RUNX2 reduced the ubiquitination level of c-Myc (Fig. 3C). To further investigate whether RUNX2 stabilized c-Myc through an interaction, exogenous RUNX2-Flag and/or c-Myc-HA in 293T cells were performed by Co-IP and the results indicated that there was an interaction between the two proteins (Fig. 3D). In agreement with this, overexpression of RUNX2 reduced c-Myc ubiquitination in T-47D cells while RUNX2 knockdown increased c-Myc ubiquitination in MDA-MB-231 cells (Fig. 3E). In parallel, endogenous RUNX2 and/or c-Myc were analyzed by Co-IP in MDA-MB-231 and 4T1 cells and the results confirmed that there was an interaction between the two proteins in breast cancer cells (Fig. 3F). A His pull-down assay showed that RUNX2 directly bound to c-Myc in vitro (Fig. 3G). These results demonstrate that RUNX2 interacts with c-Myc directly in breast cancer cells. Domain organizations of both RUNX2 and c-Myc have been extensively mapped. The protein sequence of c-Myc contains several evolutionarily conserved segments, including the so-called Myc homology boxes (MBs), which are involved in highly dynamic protein–protein interactions with a wide number of cofactors that regulate protein stability [21]. RUNX2 contains a highly conserved Runt-homology domain (RHD) that is responsible for DNA and cofactor binding, an N-terminal glutamine–alanine domain, and a C-terminal domain that interacts with various proteins in a context-dependent manner [22]. To identify the domains of RUNX2 and c-Myc responsible for their interaction, we co-expressed Flag-tagged RUNX2 deletion mutants with full-length c-Myc in 293T cells. Co-IP analysis demonstrated RUNX2 mutant lacking RHD failed to bind to c-Myc (Fig. 3H). Reciprocally, c-Myc mutants with deletions of MB domains were also co-expressed with full-length RUNX2 in 293T cells. c-Myc mutants with deletions of MB2 or MB3 were unaffected in their ability to pull down RUNX2. In contrast, the c-Myc mutant lacking the MB1 domain failed to bind to RUNX2 (Fig. 3I). Taken together, these results suggest that RUNX2 mitigates the ubiquitination of c-Myc by directly binding to the MB1 domain of c-Myc, relying on its RHD.

### RUNX2 disrupts FBXW7-mediated c-Myc ubiquitination and degradation

**Fig. 4:**
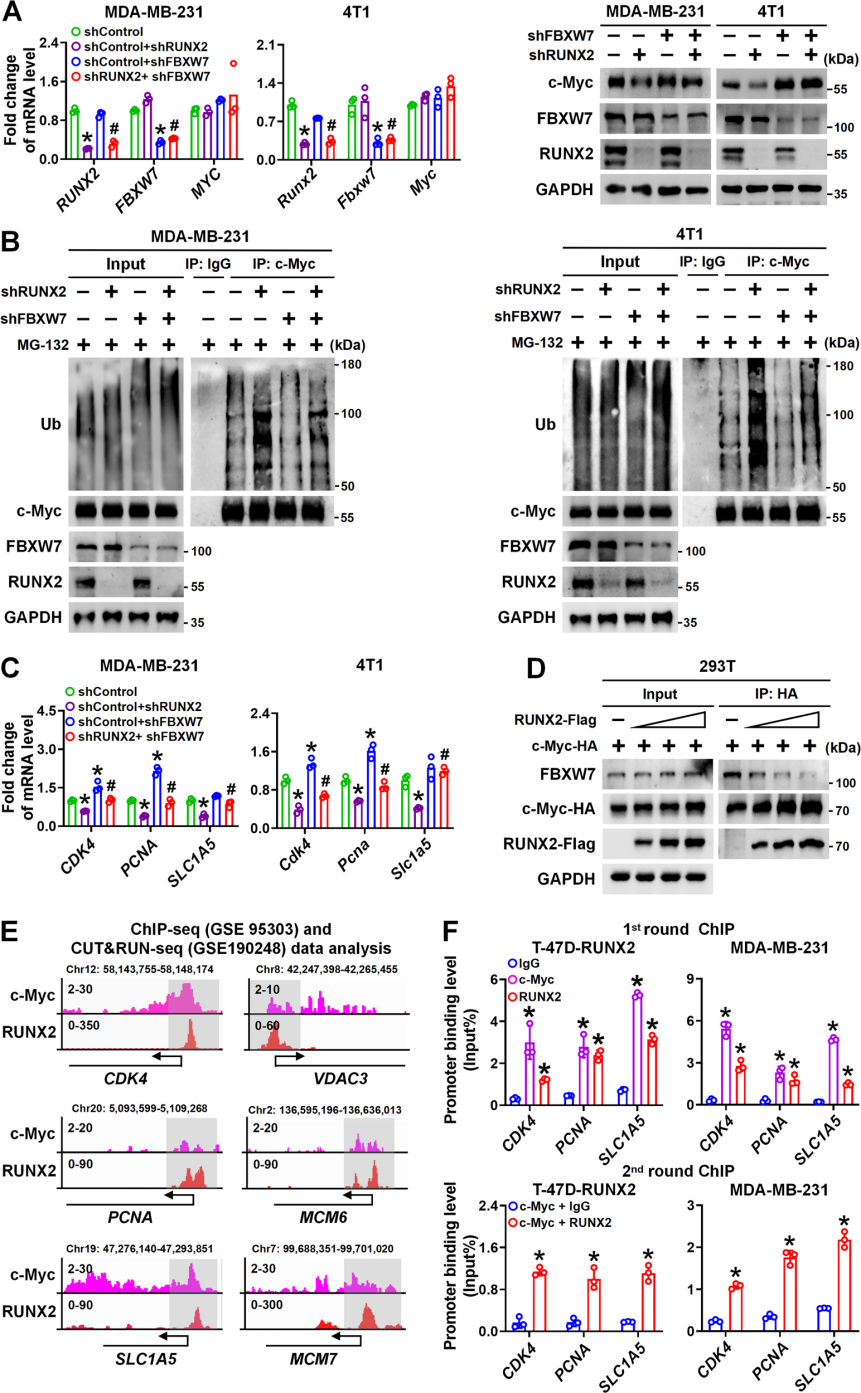
RUNX2 disrupts FBXW7-mediated c-Myc ubiquitination and degradation. **A** RUNX2 and/or FBXW7 was knocked down with lentiviral infection in MDA-MB-231 and 4T1 cells. The mRNA and protein levels of c-Myc, FBXW7, and RUNX2 in the indicated cells were detected by RT–qPCR and immunoblot. **B** c-Myc ubiquitination levels in the indicated cells were detected by IP and immunoblot following MG-132 treatment. **C** mRNA levels of c-Myc target genes in the indicated cells were detected by RT–qPCR. **D** HA-tagged c-Myc and a gradient dose of Flag-tagged RUNX2 or vector control were co-transfected into 293T cells. The protein levels of RUNX2 and FBXW7 interacting with c-Myc were detected by Co-IP and immunoblot. **E** Density maps for ChIP–seq (GSE190248) and CUT&RUN–seq (GSE95303) data showing c-Myc target genes genomic locus enriched by c-Myc and RUNX2 in MDA-MB-231 cells. The genome browser map is displayed using the IGV software. **F** Enrichment of c-Myc and RUNX2 at the promoter regions of c-Myc target genes in T-47D cells with RUNX2 overexpression and MDA-MB-231 cells was detected by Re-ChIP–qPCR analysis. Data are shown as the mean ± SD. Statistical analyses were performed with the unpaired Student’s *t*-test. *P < 0.05 compared with control cells. ^**#**^*P* < 0.05 shFBXW7 plus shRUNX2 compared with shRUNX2 cells, or shFBXW7 plus shRUNX2 compared with shFBXW7 cells. IP immunoprecipitation, ChIP–seq chromatin immunoprecipitation–sequencing.

Proteasome degradation of c-Myc is regulated by several ubiquitin ligases, including FBXW7, SKP2, HUWE1, and STUB1 [23]. We conducted a siRNA-mediated screen to identify potential ubiquitin ligases of c-Myc modulated by RUNX2. Knockdown of all four ubiquitin ligases did not alter *MYC* mRNA level (Fig. S5A). Notably, FBXW7 knockdown dramatically rescued c-Myc protein level in RUNX2-knockdown MDA-MB-231 cells, while the knockdown of SKP2, HUWE1, and STUB1 had no such effect (Fig. S5B). For further validation, RUNX2 was knocked down in MDA-MB-231 or 4T1 cells stably expressing shFBXW7 or shControl. Indeed, the knockdown of RUNX2 decreased the c-Myc protein level by enhancing its ubiquitination, while the knockdown of FBXW7 reversed this effect (Fig. 4A, B). Consistently, FBXW7 knockdown partially rescued the mRNA expression of c-Myc target genes, including *CDK4/Cdk4, PCNA/Pcna,* and *SLC1A5/Slc1a5*, which were reduced by RUNX2 depletion (Fig. 4C). Furthermore, Co-IP result revealed that RUNX2 reduced the binding of FBXW7 and c-Myc in a dose-dependent manner in 293T cells (Fig. 4D).

To determine whether c-Myc target genes are transcriptionally regulated by RUNX2/c-Myc complex, we simultaneously analyzed the datasets of c-Myc ChIP–seq and RUNX2 CUN&RUN–seq in MDA-MB-231 cells. Significantly, the peaks of c-Myc ChIP–seq and RUNX2 CUN&RUN–seq overlapped significantly on the promoter regions of c-Myc target genes (Fig. 4E). We then performed Re-ChIP–qPCR analyses in T-47D cells with RUNX2 overexpression and in MDA-MB-231 cells. As expected, the promoter regions containing c-Myc-responsive consensus motifs in *CDK4*, *PCNA*, and *SLC1A5* were significantly enriched by both c-Myc and RUNX2 in the first round ChIP–qPCR and the c-Myc enrichments could be further amplified from the Re-ChIP using a RUNX2–specific antibody, indicating that RUNX2 and c-Myc co-occupy these promoter regions (Fig. 4F). Collectively, these results demonstrate that RUNX2 hijacks c-Myc from the FBXW7/c-Myc degradation complex and forms a RUNX2/c-Myc transcriptional complex, thereby potentiating the expression of c-Myc target genes.

### c-Myc mediates RUNX2-regulated aggressiveness in breast cancer cells in vitro

**Fig. 5:**
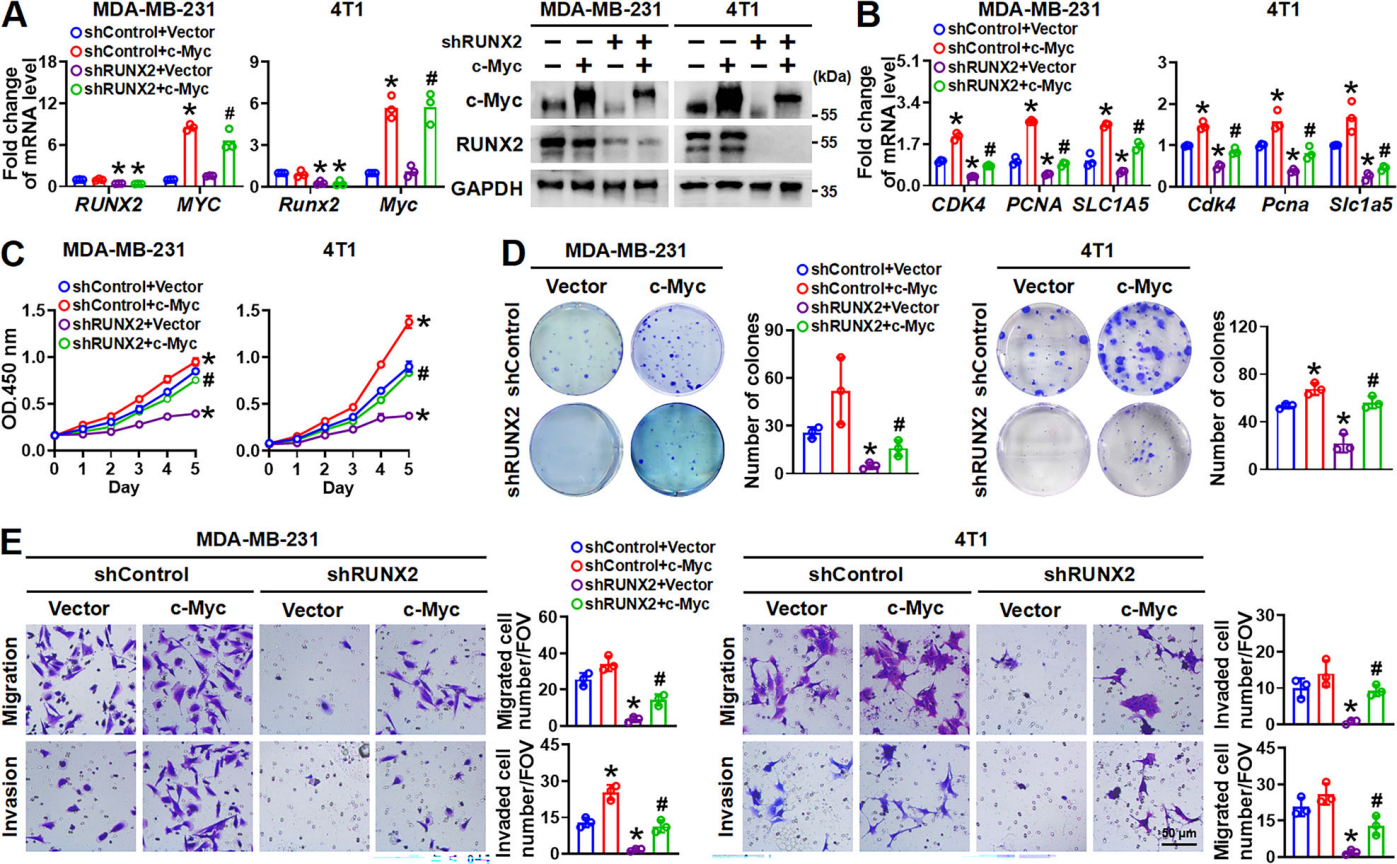
c-Myc mediates RUNX2-regulated breast cancer cell aggressiveness in vitro. **A** The mRNA and protein levels of RUNX2 and c-Myc in the indicated cells were detected by RT–qPCR and immunoblot. **B** The mRNA levels of c-Myc target genes in the indicated cells were measured by RT–qPCR. Proliferation of the indicated cells was assessed by CCK-8 assay (**C**) or colony formation assay (**D**). **E** Migration and invasion of the indicated cells were measured using Transwell assays. Data are shown as mean ± SD. Statistical analyses were performed with the unpaired Student’s *t*-test (**A**, **B**, **D**, and **E**) or two-way ANOVA (**C**). *P < 0.05 compared with control cells. ^**#**^*P* < 0.05 shRUNX2 plus c-Myc compared with shRUNX2 cells.

To further investigate whether c-Myc mediates RUNX2-regulated aggressiveness in breast cancer cells, MDA-MB-231 and 4T1 cells stably expressing c-Myc, shRUNX2, or shRUNX2 combining with c-Myc were constructed using lentiviral infection. Clearly, overexpression of c-Myc had no significant impact on RUNX2, both in the mRNA and protein level, whereas knockdown of RUNX2 decreased both exogenous and endogenous c-Myc protein levels (Fig. 5A). Furthermore, the downregulation of c-Myc target genes, including *CDK4/Cdk4*, *PCNA/Pcna* and *SLC1A5/Slc1a5* resulting from RUNX2 knockdown was rescued by the presence of c-Myc (Fig. 5B). Additionally, RUNX2 knockdown-impaired cell proliferation, colony formation, migration, and invasion abilities were restored upon c-Myc overexpression (Fig. 5C–E). These findings suggest c-Myc mediates RUNX2-regulated aggressiveness in breast cancer cells.

### c-Myc mediates RUNX2-driven breast cancer growth and multi-organ metastasis in vivo

**Fig. 6:**
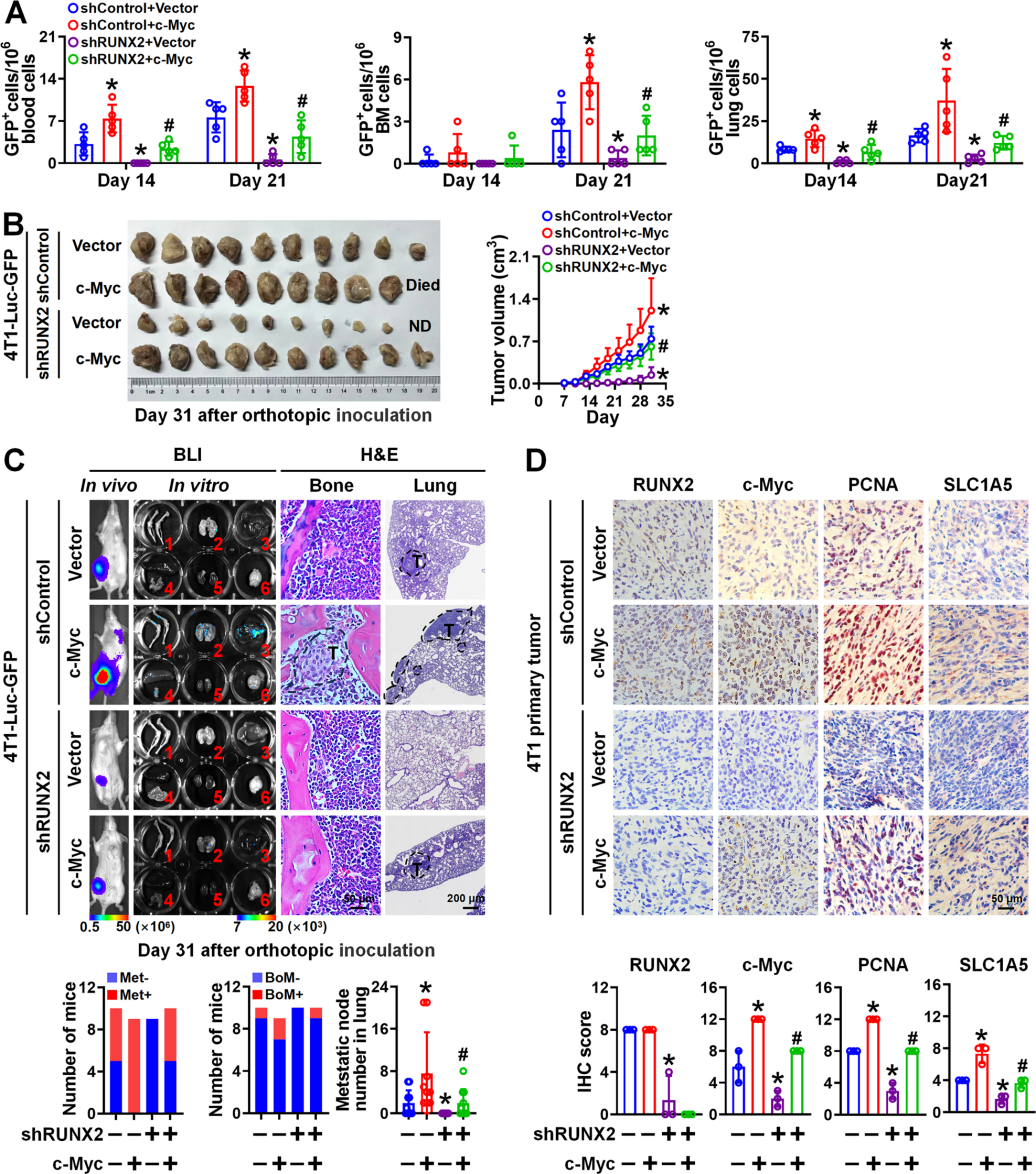
c-Myc contributes to RUNX2-mediated BC growth, dissemination, and widespread metastasis. A total of 1 × 10^5^ 4T1-Luc-GFP cells with c-Myc overexpression, RUNX2 knockdown, c-Myc overexpression combing with RUNX2 knockdown, or corresponding control were orthotopically inoculated into BALB/c mice (*n* = 10 per group). **A** The number of GFP-positive cancer cells in peripheral blood, bone marrow, and lungs of the mice (*n* = 5 per group) were counted at day 14 and day 21 using flow cytometric analysis. **B** Tumor growth was monitored and quantified. All mice were sacrificed on day 31, and the tumors dissected from the mice are shown. **C** Representative in vivo and in vitro organ BLI (1 = bone, 2 = lung, 3 = liver, 4 and 5 = spleen, diaphragm, spleen and kidney, and 6 = brain) as well as H&E staining images of the bones and lungs from the indicated mice are shown. The number of mice with metastasis, BoM, and LuM nodes were counted in each group. **D** RUNX2, c-Myc, PCNA, and SLC1A5 expression in the primary tumors harvested from the indicated mice were detected and quantified by IHC. Representative IHC images of each group are shown. Data are shown as mean ± SD. Statistical analyses were performed with the unpaired Student’s *t*-test (**A**, **C**, and **D**) or two-way ANOVA (**B**). *P < 0.05 compared with control cells. ^**#**^*P* < 0.05 shRUNX2 plus c-Myc compared with shRUNX2 cells. BLI bioluminescence imaging, Met metastasis, BoM bone metastasis.

To further investigate whether c-Myc mediates RUNX2-driven breast cancer growth and multi-organ metastasis, 4T1-Luc-GFP cells stably expressing c-Myc, shRUNX2, or c-Myc combing with shRUNX2 were orthotopically inoculated into BALB/c mice. Firstly, GFP-labeled circulating tumor cells (CTCs) in the blood, as well as disseminated tumor cells (DTCs) in the bones and lungs, were quantified using flow cytometry at day 14 and day 21 post-inoculation. The results indicated that c-Myc overexpression promoted and RUNX2 knockdown inhibited tumor cell dissemination to the circulation of blood, bone, and lung. Notably, c-Myc overexpression in RUNX2-knockdown cells rescued this inhibitory effect (Figs. 6A and S6). Additionally, c-Myc overexpression restored tumor growth and multi-organ metastasis in RUNX2-knockdown cells (Fig. 6B, C). The protein levels of RUNX2, c-Myc, PCNA, and SLC1A5 were consistent with the phenotypes observed in vitro and in clinical samples (Fig. 6D). Collectively, these results indicate that RUNX2 promotes breast cancer growth, multi-organ dissemination, and widespread metastasis in a c-Myc–dependent manner.

### RUNX2–c-Myc regulatory axis is highly active in metastatic breast cancer cells and predicts poor outcome of patients

**Fig. 7:**
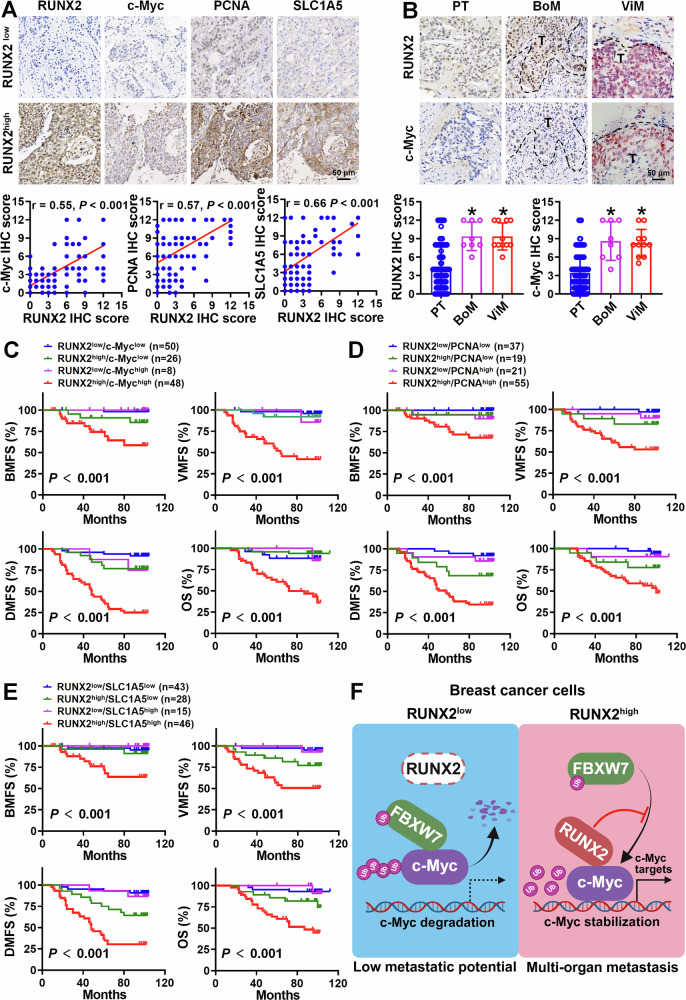
RUNX2/c-Myc regulatory axis is highly active in metastatic breast cancer cells and predicts poor outcome of patients. **A** RUNX2, c-Myc, PCNA, and SLC1A5 protein levels were detected by IHC and semi-quantified using IHC score in 132 primary breast cancer tissues. The correlation between RUNX2 and c-Myc, PCNA, or SLC1A5 protein levels was analyzed, and representative images are shown. Statistical analyses were performed using Pearson’s correlation analysis. **B** RUNX2 and c-Myc protein levels in bone metastatic (*n* = 8) and visceral-metastatic (*n* = 11) breast cancer tissues were detected by IHC and semi-quantified using the IHC score. The IHC score of RUNX2 and c-Myc in primary, bone metastatic, and visceral-metastatic breast cancer tissues were analyzed, and representative images are shown. Statistical analyses were performed with the unpaired Student’s *t*-test. Data are shown as mean ± SD. *P < 0.05 compared with primary breast cancer tissues. **C**–**E** Kaplan–Meier survival analyses of BMFS, NBMFS, DMFS, and OS in breast cancer patients based on the combined level of RUNX2 and c-Myc, PCNA, or SLC1A5 proteins in primary tumors. *P*-values were obtained using log rank test. **F** The graphical abstract illustrates RUNX2 engaging c-Myc to promote breast cancer multi-organ metastasis by preventing FBXW7-mediated ubiquitination and degradation of c-Myc. IHC immunohistochemistry, PT primary tumor, BoM bone metastasis, ViM visceral metastasis, BMFS bone metastasis-free survival, VMFS visceral metastasis-free survival, DMFS distant metastasis-free survival, OS overall survival.

To investigate the protein level correlation between RUNX2 and c-Myc or its targets PCNA and SLC1A5 in human breast cancers and the clinical relevance of their combined expression to patients’ prognosis, IHC staining was conducted, and the IHC score was assessed in 132 primary breast cancer tissue specimens. A positive correlation was observed between RUNX2 and c-Myc, PCNA or SLC1A5 protein levels in these primary breast cancer tissues (Fig. 7A). We also detected the protein levels of RUNX2 and c-Myc in 8 bone metastatic and 11 visceral-metastatic breast cancer tissue specimens, and the result revealed that both RUNX2 and c-Myc protein levels were significantly higher in breast cancer cells from bone and visceral metastasis tissues compared to those in primary tumor (Fig. 7B). In addition, patients with high RUNX2 and c-Myc, PCNA, or SLC1A5 protein levels (RUNX2^high^/c-Myc^high^, RUNX2^high^/PCNA^high^, RUNX2^high^/SLC1A5^high^) had shorter bone metastasis-free survival (BMFS), visceral metastasis-free survival (VMFS), distant metastasis-free survival (DMFS) and overall survival (OS) than other patient groups (Fig. 7C–E). These results demonstrate that RUNX2–c-Myc regulatory axis drives breast cancer multi-organ metastasis and may serve as a prognostic indicator and therapeutic target for breast cancer patients.

## Discussion

Multi-organ metastasis is the most serious event leading to an unfavorable clinical outcome in breast cancer patients. There is currently no effective therapeutic strategy for this fatal disease. Therefore, unraveling the regulatory mechanisms underlying systemic metastasis is crucial for developing predictive markers and therapeutic targets to improve the outcome of patients. In the present study, we find that RUNX2 acts as a driver of multi-organ metastasis in both ER+/LumBC and TNBC/BLBC in multiple mouse models, including orthotopic xenograft and allograft tumor models as well as hematogenous metastasis models via ventricular and tail-vein inoculations. RUNX2 expression at high levels in tumor cells increases the risk of hematogenous dissemination and multi-organ metastasis in breast cancer patients.

It has long been recognized that RUNX2 is aberrantly overexpressed in breast cancers facilities metastasis to the bone environment, however, the clinical and experimental evidence for illustrating the role of RUNX2 in bone-specific metastasis of breast cancers remains insufficient [17, 24, 25]. RUNX2 overexpression has been known to activate a cohort of genes involved in bone-remodulation, angiogenesis, cell growth, EMT, and invasion, and also confers stemness, aggressiveness, and chemoresistance phenotypes in both ER+ breast cancer [26] and TNBC [27–30]. High level of RUNX2 constitutes a major driving force in tumor progression and aggressiveness through reciprocal activation with the PI3K/AKT signaling pathway [31]. The PI3K/Akt signaling pathway plays a key role in brain metastasis of breast cancer through promoting cell proliferation, survival, metabolism, angiogenesis, and immunosuppression [32, 33]. It has been reported that the PI3K pathway is activated in most breast cancer brain metastasis regardless of subtype [34]. Additionally, RUNX2/ER complex stimulates the transcription of the stem cell factor SOX9, which is sufficient to promote proliferation, endocrine resistance, chemoresistance, immunosuppression, and metastasis of breast cancer cells [26, 35, 36]. These pieces of evidence support that RUNX2 acts as an upstream regulator of PI3K/Akt and SOX9 oncogenic transcriptions, with functions beyond driving bone-specific metastasis in breast cancer [37].

Furthermore, we found that, using the Runt-homology domain, RUNX2 prevents FBXW7-mediated ubiquitination and degradation of c-Myc through a direct interaction with c-Myc, and coordinately activates c-Myc target gene transcription and expression. In addition to function as a lineage-determining transcription factor, RUNX2 has been identified as a multifunctional modulator through protein–protein interaction that enhances the stability and/or transcriptional activity of its binding partners. RUNX2, using sites within the Runt-homology domain, physically interacts with and stabilizes HIF1-α protein, which upregulates multiple targets involved in angiogenesis and metabolism, including VEGFA, GLUT1, ENO1, and ALDOC in mesenchymal cells, pre-osteoblast, and growth plate hypertrophic chondroblasts [38, 39]. HIF1-α-mediated aberrant angiogenesis and tumor cell metabolism reprogramming are well-documented to drive breast cancer progression and metastasis [40]. RUNX2 also physically interacts with androgen receptor (AR), co-occupying an enhancer element of prolactin-induced protein (*PIP*) to synergistically stimulate its transcription, which in turn promotes the transcription of multiple AR target genes and enhances serum growth factors or dihydrotestosterone-induced proliferation in breast cancer cells [41]. In addition, RUNX2 interacts with the serine- and methionine-rich domain of monocytic leukemia zinc finger protein MOZ and MORF (MOZ-related factor). MOZ and MORF potentiate the transcriptional activity of RUNX2, and RUNX2 in turn negatively regulates the transcriptional activity of MOZ and MORF, forming a regulatory loop [42]. Interestingly, c-Myc was also found to occupy the genomic region of RUNX2 target genes (data not shown), suggesting a possible cooperative role in regulating their shared targets. Moreover, considering RUNX2 recruits NuRD complex to exert transcriptional co-repressor function by altering histone modifications [17], whether the RUNX2/c-Myc complex engages in epigenetic modulation to control the transcription of their targets requires future investigation.

c-Myc protein level is tightly regulated by ubiquitin-specific-processing proteases (USPs) and E3 ubiquitin-protein ligases. Deubiquitylation-mediated regulation of c-Myc stability is a critical mechanism for promoting proliferation and metastasis in multiple cancer types. USP5, USP17, and USP43 deubiquitinate and stabilize c-Myc to promote cancer cell proliferation and metastasis through reprogramming of glucose metabolism [43–45]. Phosphorylated ANXA2 (Tyr23) interacts with and inhibits ubiquitin-dependent proteasomal degradation of c-Myc, which potentiates HIF1-α transcription, further facilitating esophageal cancer growth and lung metastasis [46]. CircECE1 interacts with c-Myc to prevent SPOP-mediated c-Myc ubiquitination and degradation, subsequently regulating the Warburg effect and promoting osteosarcoma growth and lung metastasis [47]. CircCFL1 acts as a scaffold to enhance the interaction between HDAC1 and c-Myc, thereby sustaining the stability of c-Myc via deacetylation-mediated inhibition of K48-linked ubiquitylation, promoting growth and lung metastasis in TNBC cells with TP53 mutations [48].

FBXW7 is a substrate recognition component of an SCF (SKP1-CUL1-F-box protein) E3 ubiquitin-protein ligase complex, which mediates the phosphorylation-dependent ubiquitination and subsequent proteasomal degradation of target proteins. It has been known that FBXW7 interacts with and thereby destabilizes c-Myc in a manner dependent on phosphorylation of threonine-58 and serine-62 within the MB1 domain [49]. DNA polymerase POLD1 attenuates FBXW7-mediated ubiquitination and degradation of c-Myc by directly binding to the c-Myc MB1 domain competitively with FBXW7. The POLD1/c-Myc complex promotes the transcriptional activity of c-Myc. In turn, c-Myc increases expression of POLD1, forming a POLD1–c-Myc positive feedback loop to promote proliferation and metastasis of bladder cancer [50]. Similarly, pleckstrin-2 interacts with c-Myc and reduces ubiquitination and degradation by FBXW7, and c-Myc also directly binds to the pleckstrin-2 promoter and activates its transcription, which sustains the aggressiveness of head and neck squamous cell carcinoma [51]. In the present study, we identified RUNX2 as a competitor of FBXW7, using its Runt-homology domain to bind the MB1 domain of c-Myc, thereby hijacking c-Myc to govern synchronous multi-organ metastasis in breast cancers. As c-Myc does not regulate RUNX2 expression, RUNX2 is considered an upstream modulator of c-Myc expression, which is supported by aged MMTV-*Runx2* transgenic mice that shows elevated c-Myc protein level compared to control mice in the mammary gland hyperplasia [16].

In conclusion, the present study demonstrates that overexpression of RUNX2 promotes synchronous multi-organ metastasis rather than bone-preferred metastasis in breast cancer regardless of subtype. Mechanistically, RUNX2 hijacks c-Myc from FBXW7/c-Myc degradation complex to form a RUNX2/c-Myc transcriptional complex, thereby enhancing the expression of c-Myc target genes to drive multi-organ metastasis of breast cancer (Fig. 7F). Our findings highlight that the RUNX2–c-Myc axis may serve as an indicator and a critical target for the prediction and treatment of multi-organ metastatic breast cancer.

## Supplementary information

Supplementary Table 1

Supplementary Table 2

Supplementary Table 3

Supplementary Table 4

Supplementary Figure 1

Supplementary Figure 2

Supplementary Figure 3

Supplementary Figure 4

Supplementary Figure 5

Supplementary Figure 7

Supplementary Figure Legends

## Data Availability

All the data in this study are available from the corresponding author on reasonable request. All data generated or analyzed in this study are included within the article and its Supplementary Materials.
