# Supplementary material for: Overexpression of RUNX2 promotes breast cancer multi-organ metastasis through stabilizing c-Myc
Source: Cell Death Dis. 2025 Oct 6;16(1):696. doi: 10.1038/s41419-025-08018-9 (PMC12501288; doi:10.1038/s41419-025-08018-9)
Supplement: Supplementary file 1 — Supplementary Table 1 [file 41419_2025_8018_MOESM1_ESM.docx]

**Supplementary Table 1. Sequences of shRNAs and siRNAs**

| **shRNA/siRNA** | **Sequence** |
| --- | --- |
| shRUNX2#1 (human and mouse) | CAAATTTGCCTAACCAGAATG |
| shRUNX2#2 (human and mouse) | GAGTTTCACCTTGACCATAAC |
| shFBXW7 (human) | CCAGTCGTTAACAAGTGGAAT |
| shFBXW7 (mouse) | CAGCACAGAATTGATACAAAC |
| shControl | TTCTCCGAACGTGTCACGT |
| siFBXW7 | CAUUGAUAGUUGUGAACCA |
| siSTUB1 | CUCAGUCGGUGCUAUGAUA |
| siSKP2 | CUCAACUUUGGAGAUGAUA |
| siHUWE1 | GGCUUCCUCUGAUAUGCAA |
| siControl | UUCUCCGAACGUGUCACGU |
