# Supplementary material for: Overexpression of RUNX2 promotes breast cancer multi-organ metastasis through stabilizing c-Myc
Source: Cell Death Dis. 2025 Oct 6;16(1):696. doi: 10.1038/s41419-025-08018-9 (PMC12501288; doi:10.1038/s41419-025-08018-9)
Supplement: Supplementary file 2 — Supplementary Table 2 [file 41419_2025_8018_MOESM2_ESM.docx]

**Supplementary Table 2. Sequences of primers for RT‒qPCR assays**

| **Gene** | **Forward primer** | **Reverse primer** |
| --- | --- | --- |
| *RUNX2/Runx2* | CTCTCACCAAGTCCTTTTAATC | AGGAGGGGTAAGATGGTCATAG |
| *GAPDH* | ACAACTTTGGTATCGTGGAAGG | GCCATCACGCCACAGTTTC |
| *Gapdh* | ATTGTCAGCAATGCATCCTG | ATGGACTGTGGTCATGAGCC |
| *CDK4* | GGAAACTCTGAAGCCGACCA | CTCCCGACTCCTCCATCTCA |
| *Cdk4* | GGCTGATGGATGTCTGTGCT | CCAGGCCGCTTAGAAACTGA |
| *PCNA* | TCTGAGGGCTTCGACACCTA | CGCGTTATCTTCGGCCCTTA |
| *Pcna* | CCGTGAGTGGAGAACTGACC | GGGCATTGAGTGTGGTGAGA |
| *SLC1A5* | CTCCTTGATCCTGGCTGTGG | GGGCAGCTCACTCTTCACTT |
| *Slc1a5* | CATCAACGACTCTGTTGTAGAC | CTGGATACAGGATTGCGGTATTT |
| *MYC* | GTCACACCCTTCTCCCTTCG | CGGGTCGCAGATGAAACTCT |
| *Myc* | GCTACGTCCTTCTCCCCAAG | ATGAAGGTCTCGTCGTCAGG |
| *FBXW7* | GGGCGGTTTTGGAGAAGTTG | TGCTCATAGTAGCTCCCGGT |
| *Fbxw7* | GGTGAAGGTGTGGGATCCAG | GGTGTCCTGTTAGCGTGTGA |
| *SKP2* | GAGAAAGAGGAGCCCGACAG | GCCTGCGGACAATCACAAAG |
| *HUWE1* | CGAAACCACGAGGGTGATGA | CACACAGGTAGTCGGGGATG |
| *STUB1* | GTGCCTCCACTGAGACTTCC | CCCTTGCCTTTCTGTTTGCC |
