# Supplementary material for: Overexpression of RUNX2 promotes breast cancer multi-organ metastasis through stabilizing c-Myc
Source: Cell Death Dis. 2025 Oct 6;16(1):696. doi: 10.1038/s41419-025-08018-9 (PMC12501288; doi:10.1038/s41419-025-08018-9)
Supplement: Supplementary file 3 — Supplementary Table 3 [file 41419_2025_8018_MOESM3_ESM.docx]

**Supplementary Table 3. Antibodies used in immunoreaction-based assays**

| **Protein** | **Company** | **Catalogue**  **number** | **RRID** | **Application** |
| --- | --- | --- | --- | --- |
| **Primary antibody** | |  |  |  |
| RUNX2 | Santa Cruz | sc-390351 | AB_2892645 | IB 1:500 |
| RUNX2 | Abcam | ab236639 | AB_2937078 | IHC 1:1500  mIHC 1:1500  IP 1:100  ChIP 1:100 |
| GAPDH | Abclonal | AC001 | AB_2769570 | IB 1:10000 |
| c-Myc | Abcam | ab32072 | AB_731658 | IB 1:2000  IHC 1:400  mIHC 1:400  IP 1:100  ChIP 1:100 |
| Flag-tag | Cell Signaling | 14793 | AB_2572291 | IB 1:1000 |
| HA-tag | Abcam | ab9110 | AB_307019 | IB 1:5000 |
| His-tag | Cell Signaling | 2365 | AB_2115720 | IB 1:1000 |
| Ubiquitin | Cell Signaling | 23026 | AB_3064918 | IB 1:1000 |
| FBXW7 | Proteintech | 28424-1-AP | AB_2881138 | IB 1:1000 |
| PCNA | Cell Signaling | 13110 | AB_2636979 | mIHC 1:1000  IHC 1:1000 |
| SLC1A5 | Abclonal | A23156 | AB_3674371 | mIHC 1:500  IHC 1:500 |
| **Secondary antibody** | |  |  |  |
| Anti-Rabbit | Abclonal | AS014 | AB_2769854 | IB 1:5000 |
| Anti-Mouse | Abclonal | AS003 | AB_2769851 | IB 1:5000 |

IB, immunoblot; IHC, immunohistochemistry; mIHC, multiplex immunohistochemistry; IP, immunoprecipitation; ChIP, chromatin immunoprecipitation.
