# Supplementary material for: Overexpression of RUNX2 promotes breast cancer multi-organ metastasis through stabilizing c-Myc
Source: Cell Death Dis. 2025 Oct 6;16(1):696. doi: 10.1038/s41419-025-08018-9 (PMC12501288; doi:10.1038/s41419-025-08018-9)
Supplement: Supplementary file 4 — Supplementary Table 4 [file 41419_2025_8018_MOESM4_ESM.docx]

**Supplementary Table 4. Sequences of primers for Re-ChIP‒qPCR assays**

| **Gene** | **Region on promoter** | **Forward primer** | **Reverse primer** |
| --- | --- | --- | --- |
| *CDK4* | –301/–391 | TCAGAGCAATGTCAAGCGGT | GGAGGAGGGCGAAGAGTGTA |
| *PCNA* | –104/–257 | TTGGCCCTAAAGTCTTCCCC | CCTGCAACCGTTTAATGCCG |
| *SLC1A5* | –580/–671 | GCCCCCTACCCTTATCCCTG | GTAGCGGTTACCAGCCAGAG |
