# Supplementary figures and images for: Overexpression of RUNX2 promotes breast cancer multi-organ metastasis through stabilizing c-Myc
Source: Cell Death Dis. 2025 Oct 6;16(1):696. doi: 10.1038/s41419-025-08018-9 (PMC12501288; doi:10.1038/s41419-025-08018-9)

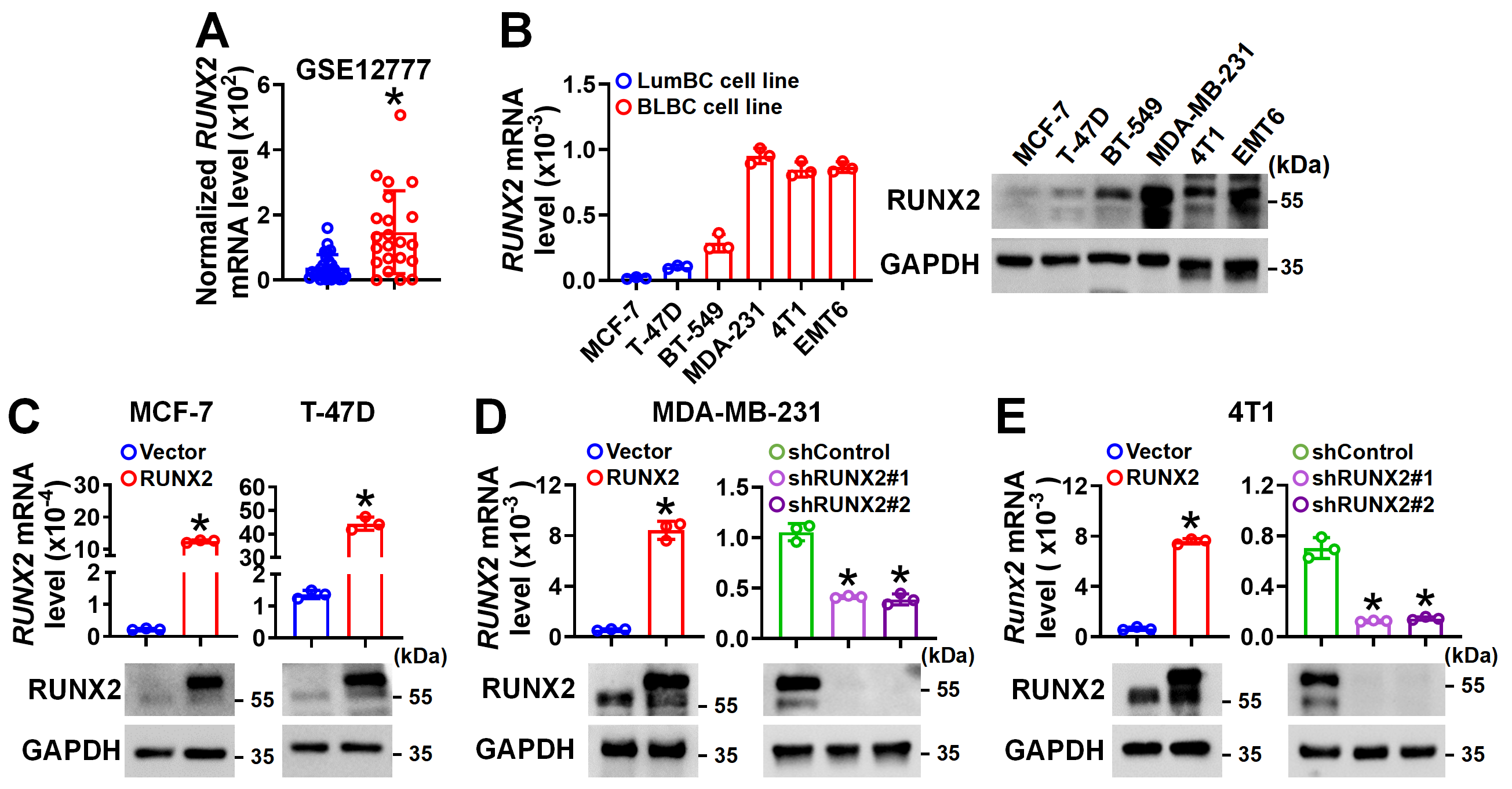

Supplement: Supplementary file 5 — Supplementary Figure 1 [file 41419_2025_8018_MOESM5_ESM.tif]

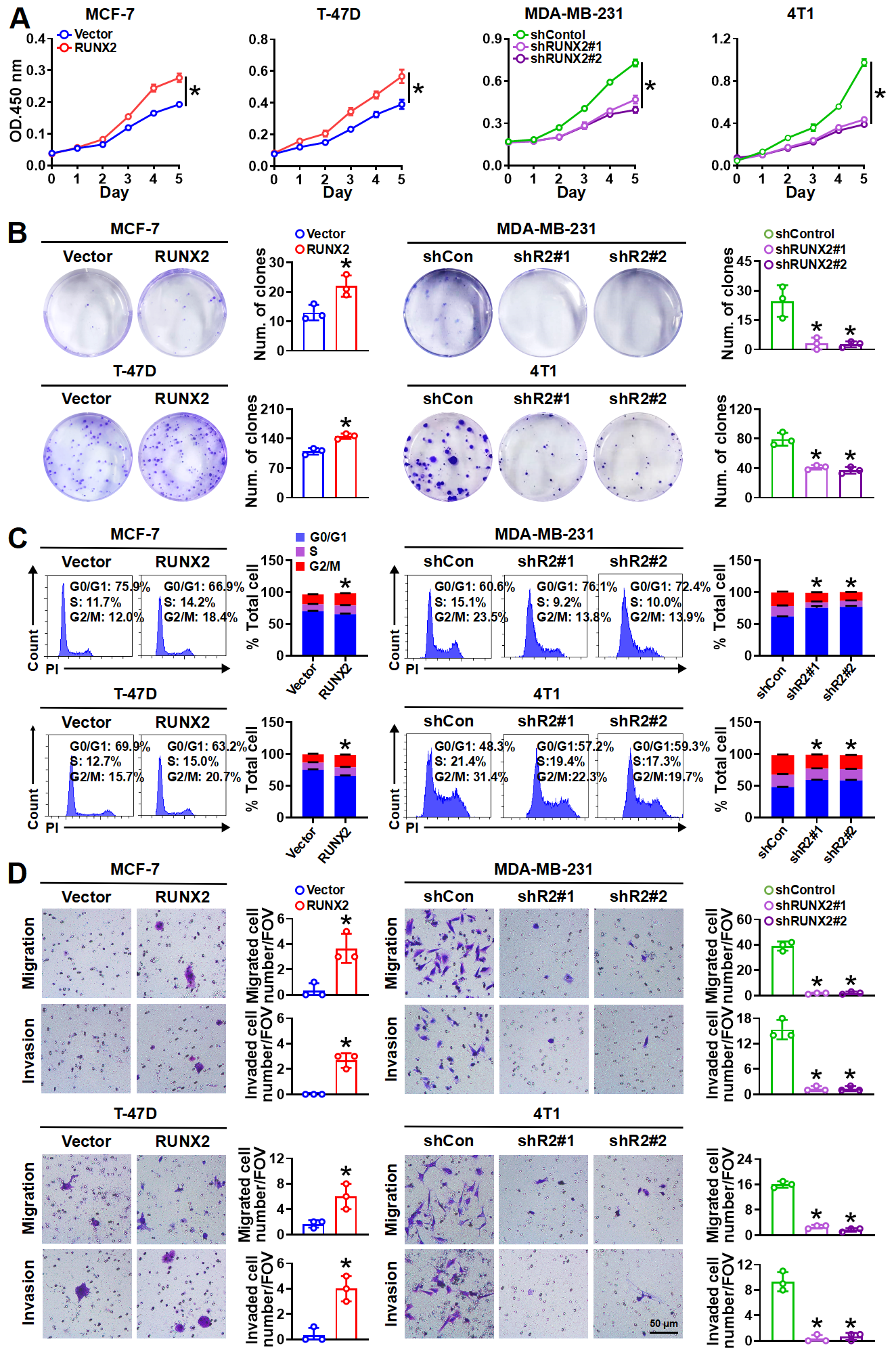

Supplement: Supplementary file 6 — Supplementary Figure 2 [file 41419_2025_8018_MOESM6_ESM.tif]

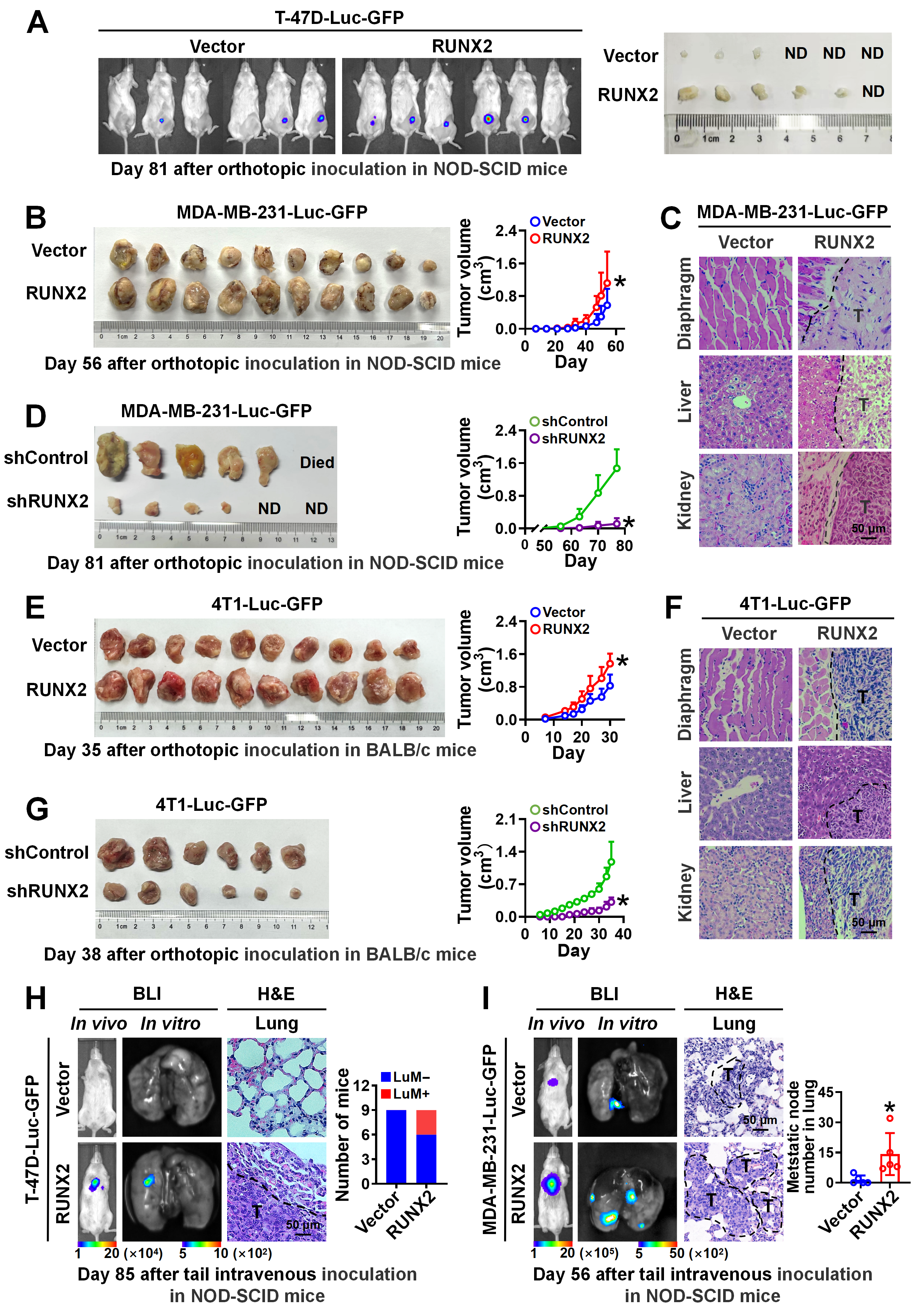

Supplement: Supplementary file 7 — Supplementary Figure 3 [file 41419_2025_8018_MOESM7_ESM.tif]

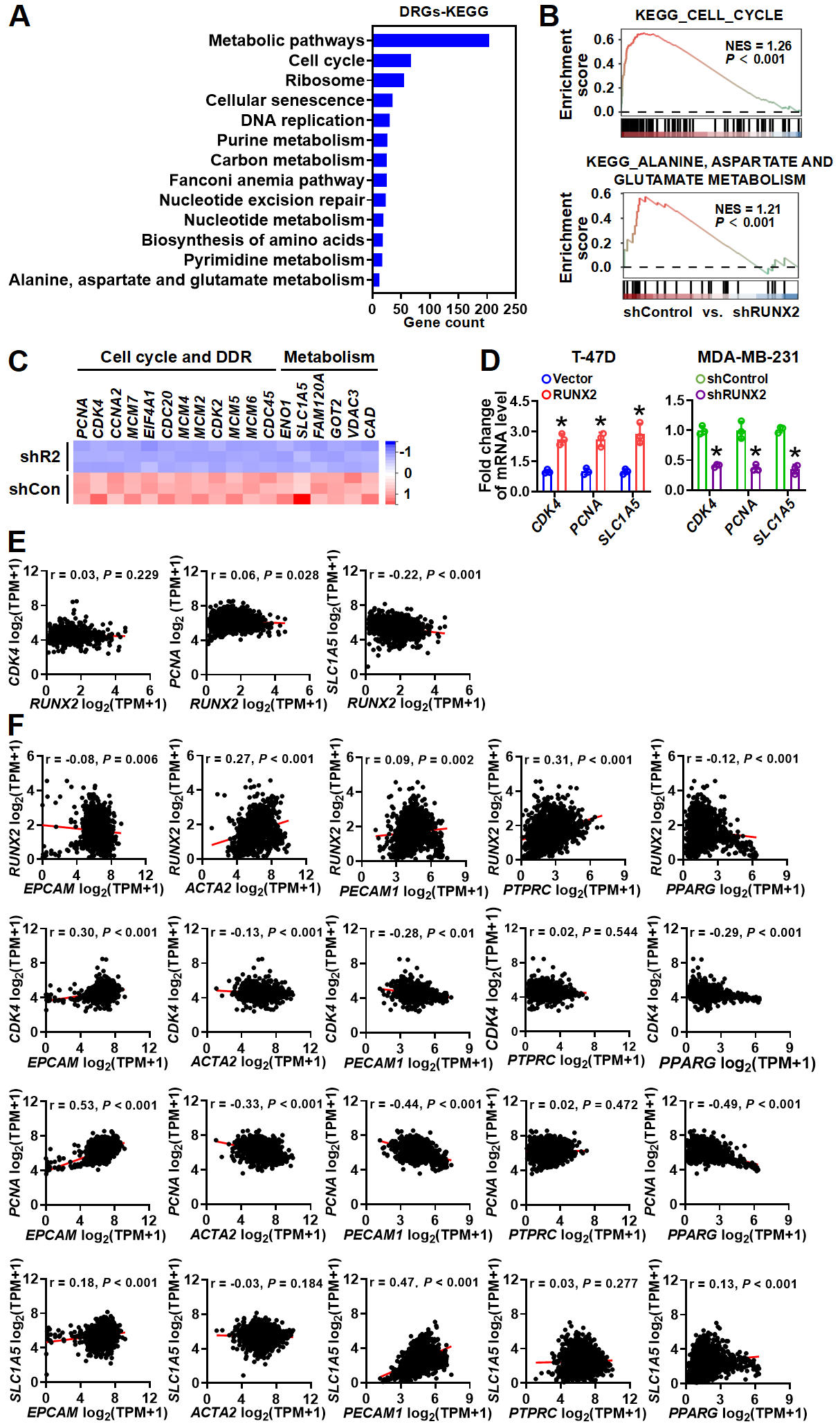

Supplement: Supplementary file 8 — Supplementary Figure 4 [file 41419_2025_8018_MOESM8_ESM.tif]

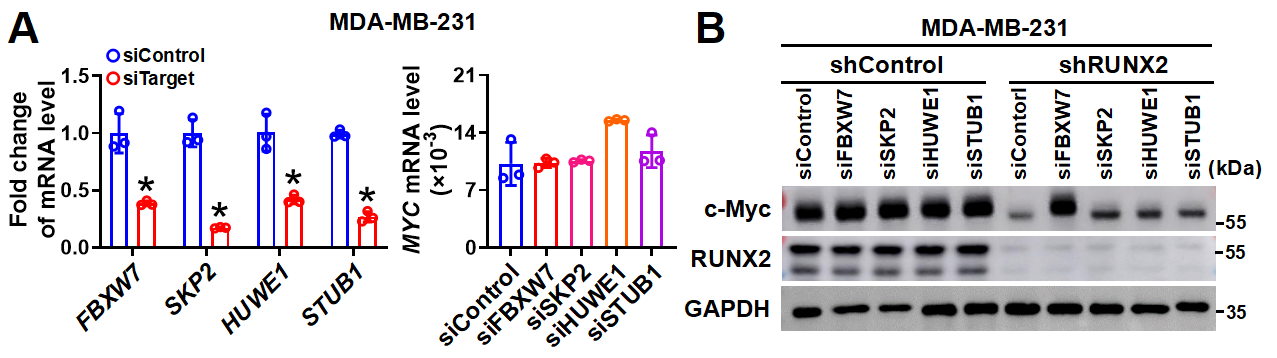

Supplement: Supplementary file 9 — Supplementary Figure 5 [file 41419_2025_8018_MOESM9_ESM.tif]

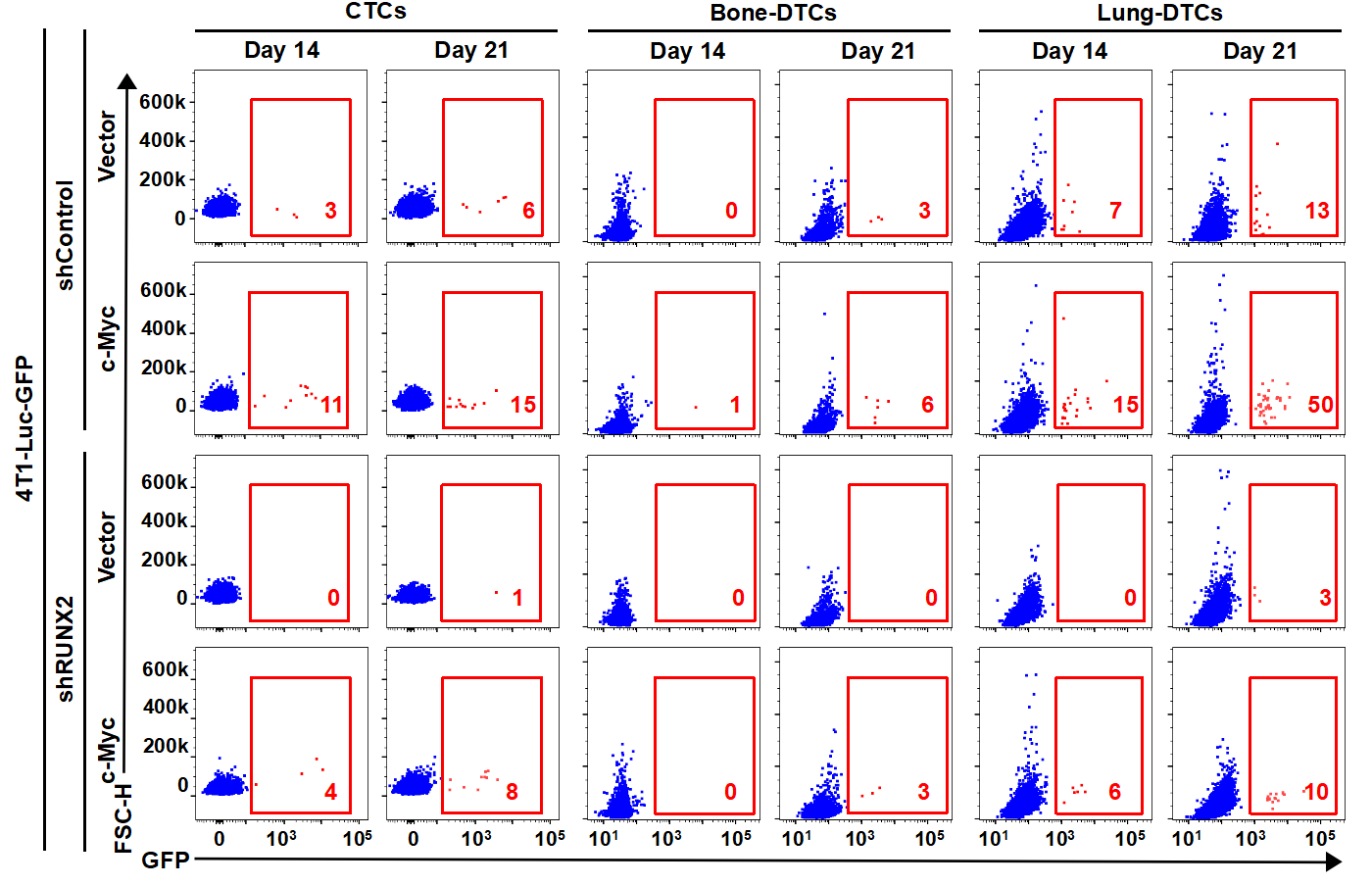

Supplement: Supplementary file 10 — Supplementary Figure 7 [file 41419_2025_8018_MOESM10_ESM.tif]
