# Supplementary material for: Overexpression of RUNX2 promotes breast cancer multi-organ metastasis through stabilizing c-Myc
Source: Cell Death Dis. 2025 Oct 6;16(1):696. doi: 10.1038/s41419-025-08018-9 (PMC12501288; doi:10.1038/s41419-025-08018-9)
Supplement: Supplementary file 11 — Supplementary Figure Legends [file 41419_2025_8018_MOESM11_ESM.docx]

**Supplemental Figure Legends**

**Fig. 1 RUNX2 mRNA and protein expression levels and the efficiency of RUNX2 overexpression and knockdown in breast cancer cell lines. A** *RUNX2* mRNA levels in the breast cancer cell lines were analyzed based on GSE12777 database. **B** RUNX2/Runx2 mRNA and protein levels were detected by RT–qPCR and immunoblot across breast cancer cell lines. **C–E** Establishment of breast cancer cells with stable overexpression or knockdown of RUNX2. The mRNA expression and protein levels of RUNX2 in the indicated cells were assessed by RT–qPCR and immunoblot. Data are presented as mean ± SD. Statistical analyses were performed with the unpaired Student’s t-test. **P* < 0.05 compared with the control cells. LumBC, luminal breast cancer; BLBC, basal-like breast cancer. Related to Fig. 1.

**Fig. 2 RUNX2 promotes breast cancer cell proliferation, migration and invasion *in vitro*.** Proliferation of the indicated cells was assessed by the CCK8 assay (**A)** or colony formation assay (**B**). **C** Cell cycle distribution of the indicated cells was analyzed via flow cytometry. **D** Migration and invasion of the indicated cells were assessed by Transwell assay. Data are shown as mean ± SD. Statistical analyses were performed with two-way ANOVA (A) or the unpaired Student’s t-test (B–D). **P* < 0.05 compared with control cells. shCon, shControl; shR2, shRUNX2; FOV, field of view. Related to Fig. 1.

**Fig. 3 RUNX2 promotes breast cancer cell growth and lung metastasis *in vivo*. A** A total of 1 × 10^7^ T-47D-Luc-GFP cells with stable RUNX2 overexpression or vector control were orthotopically inoculated into NOD-SCID mice (n = 6 per group, sacrificed on day 81). Overview of the *in vivo* BLI images and primary tumors dissected from the mice. **B**, **D**, **E** and **G** Tumor growth in the mice of Fig. 1A–D was monitored and quantified. Tumor images dissected from the indicated mice are shown. **C** and **F** Representative H&E staining of the diaphragm, liver and kidney are shown in the mice of Fig. 1A and 1C. **H** and **I** Breast cancer cells were intravenously injected into the NOD-SCID mice, including T-47D cells (1 × 10^6^ cells per mouse, n = 9 per group, sacrificed on day 85) and MDA-231 cells (1 × 10^5^ cells per mouse, n = 5 per group, sacrificed on day 56) with RUNX2 overexpression or vector control. Representative images of BLI *in vivo* and *in vitro* as well as H&E staining of the lung are shown. The number of mice with lung metastasis or the metastatic nodes was counted in each group. Data are shown as mean ± SD. Statistical analyses were performed with two-way ANOVA (B, D, E and G) or the unpaired Student’s t-test (I). **P* < 0.05 compared with the control group. ND, not detected; LuM, lung metastasis. Related to Fig. 1.

**Fig. 4 RUNX2 boosts c-Myc signaling in breast cancer cells through elevating c-Myc protein level. A** KEGG pathway analysis of downregulated genes following RUNX2 knockdown. **B** Enrichment analysis of pathways related to KEGG_CELL_CYCLE, and KEGG_ALANINE_ASPARTATE_AND_GLUTAMATE_METABOLISM. **C** Heatmap depicting downregulated c-Myc target genes in RUNX2-knockdown MDA-MB-231 cells. **D** mRNA levels of c-Myc target genes including *CDK4*, *PCNA* and *SLC1A5* in the indicated cells were measured by RT–qPCR. **P* < 0.05 compared with control cells. Pearson’s correlation analysis of the expression levels of *RUNX2* with c-Myc target genes (**E**) and *RUNX2* or c-Myc target gene with lineage markers (**F**) in breast cancer tissues based on the TCGA-BRCA dataset (n = 1089). Data are shown as mean ± SD. Statistical analyses were performed with the unpaired Student’s t test or Pearson’s correlation analysis (E and F). DRGs-KEGG, down regulated genes–Kyoto Encyclopedia of Genes and Genomes; NES, normalized enrichment score; DDR, DNA damage repair. TPM, transcripts per million. Related to Fig. 2.

**Fig. 5 FBXW7 is identified as the E3 ubiquitin ligases of c-Myc modulated by RUNX2. A** The E3 ubiquitin ligase were knocked down with siRNA transfection in MDA-231 cells. The efficiency of the knockdown and *MYC* mRNA levels were evaluated by RT–qPCR. **B** c-Myc protein levels with knockdown of the c-Myc E3 ubiquitin ligases in RUNX2 knockdown or control MDA-231 cells were detected by immunoblot. Data are shown as the mean ± SD. Statistical analyses were performed with the unpaired Student’s t-test. Related to Fig. 4.

**Fig. 6** Representative dot plots of flow cytometry for the quantification of GFP^+^ cells in the peripheral blood, bone marrow and lung tissues collected from the indicated mice shown in Fig. 6A on day 14 and day 21. CTCs, circulating tumor cells; DTCs, disseminated tumor cells.
